# Mixed-methods randomised study exploring the feasibility and acceptability of eye-movement desensitisation and reprocessing for improving the mental health of traumatised survivors of intensive care following hospital discharge: protocol

**DOI:** 10.1136/bmjopen-2023-081969

**Published:** 2024-01-29

**Authors:** Andrew Bates, Hannah Golding, Sophie Rushbrook, Julie Highfield, Natalie Pattison, David Baldwin, Michael P W Grocott, Rebecca Cusack

**Affiliations:** 1 Perioperative and Critical Care Theme, NIHR Southampton Biomedical Research Centre, University Hospital Southampton NHS Foundation Trust, Southampton, UK; 2 Clinical and Experimental Sciences, Faculty of Medicine, University of Southampton, Southampton, UK; 3 Dorset HealthCare University NHS Foundation Trust, Poole, UK; 4 Cardiff and Vale University Health Board, Cardiff, UK; 5 University of Hertfordshire, Hatfield, UK; 6 East and North Hertfordshire NHS Trust, Stevenage, UK; 7 Southern Health NHS Foundation Trust, Southampton, UK; 8 University of Southampton Faculty of Medicine, Southampton, UK

**Keywords:** PSYCHIATRY, Anxiety disorders, INTENSIVE & CRITICAL CARE, Feasibility Studies, MENTAL HEALTH

## Abstract

**Introduction:**

Post-traumatic symptoms are common among patients discharged from intensive care units (ICUs), adversely affecting well-being, increasing healthcare utilisation and delaying return to work. Non-pharmacological approaches (eg, music, therapeutic touch and patient diaries) have been suggested as candidate interventions and trauma-focused psychological interventions have been endorsed by international bodies. Neither category of intervention is supported by definitive evidence of long-term clinical effectiveness in patients who have been critically ill. This study assesses the feasibility and acceptability of using eye-movement desensitisation and reprocessing (EMDR) to improve the mental health of ICU survivors.

**Methods and analysis:**

EMERALD is a multicentre, two-part consent, pilot feasibility study, recruiting discharged ICU survivors from three hospitals in the UK. We are gathering demographics and measuring post-traumatic symptoms, anxiety, depression and quality of life at baseline. Two months after discharge, participants are screened for symptoms of post-traumatic stress disorder (PTSD) using the Impact of Events Scale-Revised (IES-R). Patients with IES-R scores<22 continue in an observation arm for 12 month follow-up. IES-R scores≥22 indicate above-threshold PTSD symptoms and trigger invitation to consent for part B: a randomised controlled trial (RCT) of EMDR versus usual care, with 1:1 randomisation. The study assesses feasibility (recruitment, retention and intervention fidelity) and acceptability (through semistructured interviews), using a theoretical acceptability framework. Clinical outcomes (PTSD, anxiety, depression and quality of life) are collected at baseline, 2 and 12 months, informing power calculations for a definitive RCT, with quantitative and qualitative data convergence guiding RCT refinements.

**Ethics and dissemination:**

This study has undergone external expert peer review and is funded by the National Institute for Health and Care Research (grant number: NIHR302160). Ethical approval has been granted by South Central-Hampshire A Research Ethics Committee (IRAS number: 317291). Results will be disseminated through the lay media, social media, peer-reviewed publication and conference presentation.

**Trial registration number:**

NCT05591625.

**STRENGTHS AND LIMITATIONS OF THIS STUDY**

- Adheres to Medical Research Council guidance for evaluating complex healthcare interventions.
- Mixed methods probe feasibility and acceptability enabling us to address cultural and contextual factors.
- Consistent with existing clinical pathways and best practice guidance.
- Not powered to detect between-group, clinically significant differences in post-traumatic symptoms.

## Introduction

### Background and rationale

Critically ill patients in intensive care units (ICUs) receive life-saving treatment, yet the burden of long-term physical, cognitive and mental health issues, collectively known as ‘postintensive care syndrome’, is significant.^1^ Global ICU admissions are on the rise^2^ and there is growing recognition of the need to address post-ICU survivorship as a defining challenge in 21st-century intensive care medicine.^3^ Despite this, healthcare providers often overlook this phase,^4^ resulting in multiple care transitions away from clinicians with an understanding of the underlying aetiology.^5^

Amidst the existential threat of critical illness, patients endure invasive treatments, potent psychoactive drugs, a busy and confusing environment and limited communication, leading to normal acute anxiety responses.^6^ However, a substantial proportion continue to suffer unpleasant psychological and somatic symptoms. Post-ICU discharge, 20%–25% experience symptoms similar to those of post-traumatic stress disorder (PTSD),^7^ with over 30% and 40% experiencing depression^8^ and anxiety,^9^ respectively. These symptoms can be persistent,^10^ co-occurring^11^ and are associated with adverse outcomes including reduced quality of life, increased healthcare utilisation and delayed return to work.^9 12 13^

Despite this, access to clinical psychology remains under-represented in UK ICU recovery services.^14^ Interventions like music therapy,^15^ therapeutic touch^16^ and patient diaries^17^ have been explored, but systematic reviews reveal that definitive evidence of long-term effect is lacking. Trauma-focused psychological therapies, such as eye movement desensitisation and reprocessing (EMDR), offer some promise, with meta-analyses showing significant reductions in PTSD, anxiety and depression for treating a diverse range of traumatised populations.^18 19^ EMDR is cost-effective^20^ and is internationally recommended by major organisations for trauma-related symptoms.^21–24^

Recent investigations of EMDR’s effectiveness in treating medical event-induced trauma, following cancer, stroke, cardiac events and multiple sclerosis have yielded promising but inconclusive findings.^25^ Case studies with ICU survivors^26 27^ and our own novel work with survivors of COVID-19-related critical illness^28^ also show promise, underscoring the need for systematic evaluation in this population. However, definitive evidence of benefit is not available.

### Objectives

The primary objective of the EMERALD study is to evaluate the feasibility and acceptability of an EMDR intervention for adult patients displaying traumatic stress symptoms following ICU discharge. These findings will guide the design of a robust, fully powered randomised controlled trial (RCT), aligning with Medical Research Council (MRC) guidance on evaluating complex medical interventions. Secondary clinical outcomes will inform the selection of a primary outcome for the larger trial and provide variance estimates for sample size calculations. Additionally, a light-touch observation arm will offer insights into the mental health trajectory of ICU survivors without traumatic stress symptoms 2 months after hospital discharge.

## Methods: participants, interventions and outcomes

### Design

**Figure 1:**
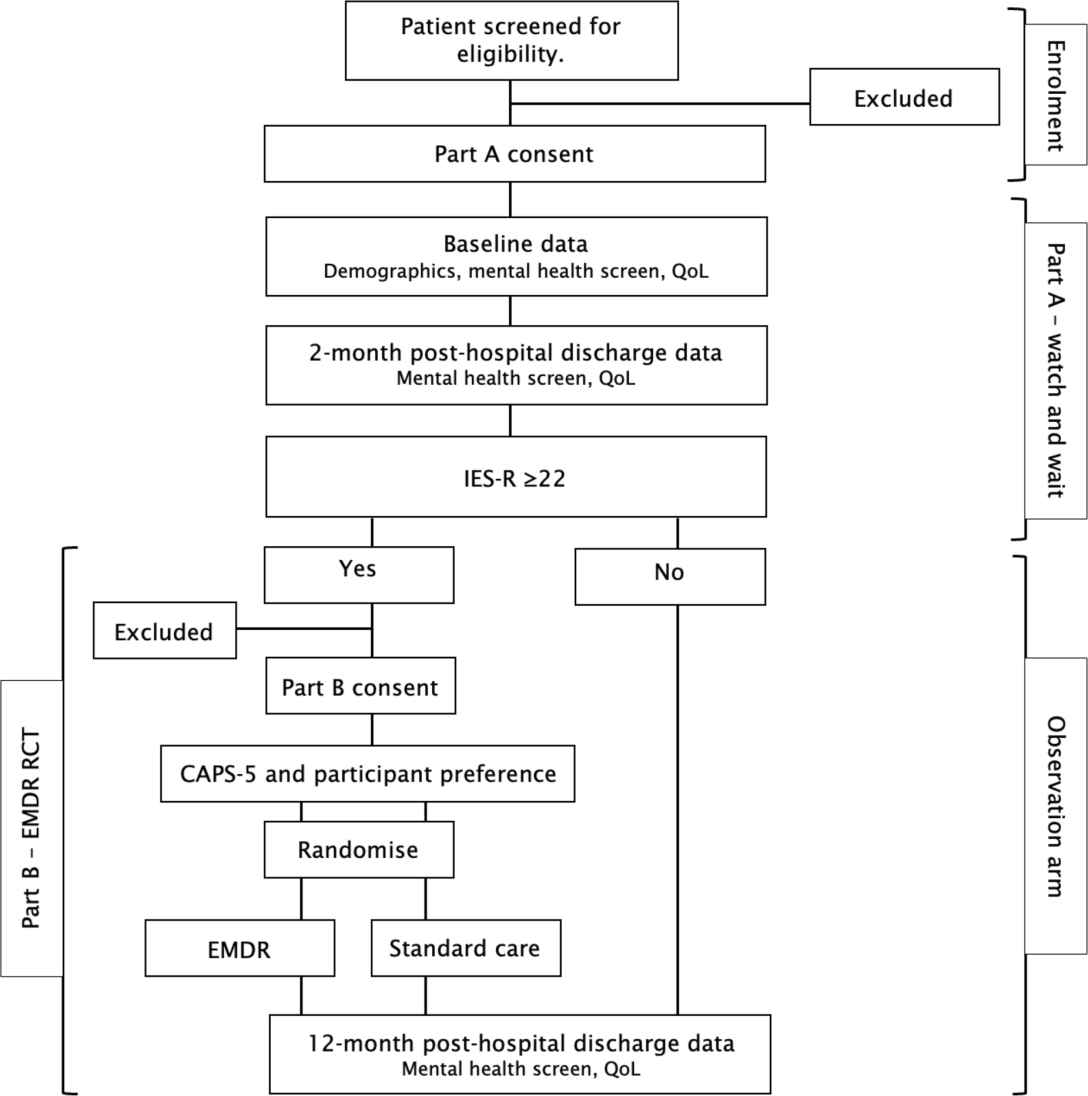
EMERALD participant timeline. CAPS-5, Clinician Administered PTSD Scale for DSM-5; EMDR, eye-movement desensitisation and reprocessing; IES-R, Impact of Events Scale-Revised; PTSD, post-traumatic stress disorder; QoL, quality of life; RCT, randomised controlled trial.

This is a multicentre, mixed-methods, randomised controlled pilot feasibility study, with a two-part consent process and is reported using the Standard Protocol Items: Recommendations for Interventional Trials (SPIRIT) reporting guidelines^29^ (online supplemental file 1: Reporting checklist for protocol of a clinical trial). Initially, all participants enter part A, which is an observational study, where they complete a series of mental health questionnaires at baseline, 2 months and 12 months posthospital discharge. If a participant shows symptoms of post-traumatic stress at the 2 month mark (scoring≥22 on the Impact Events Scale-Revised (IES-R)), they are invited to consider participating in part B, which is an interventional study of EMDR versus standard care. Those without post-traumatic stress symptoms at 2 months (≤21 on the IES-R) or those who decline participation in part B will be offered continuation of the observation arm. All participants from both part A and part B repeat the study assessments at 12 months posthospital discharge. See figure 1 for the participant timeline.

10.1136/bmjopen-2023-081969.supp1 Supplementary data

### Study setting

The study is sponsored by the University Hospital Southampton National Health Service (NHS) Foundation Trust (FT). Recruitment will occur after adult patients are discharged from three adult NHS ICUs in the UK: University Hospital Southampton, Royal Bournemouth Hospital and Poole General Hospital. The intervention will be provided through NHS psychological therapy services in proximity to the study participants, specifically Southern Health NHS FT and Dorset Healthcare University NHS FT.

### Part A participant recruitment

Recruitment is anticipated to occur between February 2023 and May 2024. Eligibility screening will target consecutive patients discharged from the participating ICUs. Research staff will approach eligible patients on hospital wards or within 2 months following hospital discharge, via a telephone call or email, providing a participant information sheet. Patients will be invited to complete an informed consent form (ICF), accessible electronically through Qualtrics on tablet devices provided by the trial team, via an emailed link or on paper to suit patient preference. This initial consent pertains to their participation in the observational study (part A), involving baseline data collection and psychometric assessments, with a follow-up evaluation at 2 months and 12 months following hospital discharge.

### Eligibility criteria

Eligibility will be determined by hospital research nurses acting under delegated authority of the local Principal Investigator. Patients will be eligible for part A if they meet the following criteria:

- Survivor of an intensive care admission, who received level 3 care for >24 hours.
- Aged≥18 years.
- Capacity to provide informed consent.

Patients will be excluded if they meet any of the following criteria:

- Pre-existing cognitive impairment such as dementia.
- Pre-existing diagnosis of psychosis.
- Not expected to survive beyond hospital discharge.
- Traumatic brain injury.

### Baseline data collection

Research staff will collect demographic data, medical history and ICU admission history following consent. All participants will complete the Impact of Events Scale-Revised (IES-R), Patient Health Questionnaire-9 (PHQ-9), Generalised Anxiety Disorder 7 (GAD-7) and the Euroqol 5 Dimension 5 Level (EQ-5D-5L).

### Two-month posthospital discharge assessment

All participants will be requested to repeat the IES-R, PHQ-9, GAD-7 and EQ-5D-5L. These patient-reported outcome measures can be completed electronically via an emailed link or by using paper versions sent with a prepaid return envelope.

The study team will review the IES-R responses. Participants with a total score≥22, indicative of post-traumatic stress symptoms, will be approached to consider participation in an EMDR versus usual care RCT (part B).

Participants without symptoms (IES-R≤21) or those not interested or unable to participate in the RCT will continue in the observational study, completing the 12 month follow-up assessment.

### 12-month follow-up assessment

Research staff will ask all participants, in both the observation group (part A only) and RCT (part A and part B), to repeat the IES-R, PHQ-9, GAD-7 and the EQ-5D-5L, at 12 months posthospital discharge. See table 1 for the full study schedule of events.

**Table 1:** EMERALD study schedule of events

	Baseline	2 months postdischarge	3–9 months postdischarge	12 months postdischarge
Informed consent	X Part A	X* Part B		
Demographics	X			
IES-R	X	X		X
CAPS-5, CGI-S*		X*		X*
PHQ-9	X	X		X
GAD-7	X	X		X
EQ-5D-5L	X	X		X
EMDR intervention			X*	
IES-R, PHQ-9, GAD-7 (EMDR group only)				
Randomisation preference*		X*		
Process evaluation			X	X

X* for participants consenting to part B of the study only.

CAPS-5, Clinician Administered PTSD Scale for DSM-5; CGI-S, Clinical Global Impression of Illness Severity; EMDR, eye-movement desensitisation and reprocessing; EQ-5D-5L, EuroQol-5 Dimensions-5 Levels; GAD-7, Generalised Anxiety Disorder 7; IES-R, Impact of Events Scale-Revised; PHQ-9, Patient Health Questionnaire-9; PTSD, post-traumatic stress disorder; QoL, quality of life; RCT, randomised controlled trial.

### Part B participant recruitment

Participants scoring≥22 on the 2 month IES-R will receive a phone call or email from the study team, inviting them to consider consenting to part B, the EMDR versus usual care RCT. The part B PIS and ICF will be accessible electronically or via postal delivery. Those who consent to part B will first undergo a Clinician Administered PTSD Scale for Diagnostic and Statistical Manual of Mental Disorders, fifth edition (CAPS-5) assessment to evaluate PTSD symptoms and a Clinical Global Impression of Illness Severity (CGI-S) assessment with the Chief Investigator (CI). Additionally, participants will be asked to rate their preference for study arm strength using a Likert scale ranging from 0 to 10.

### Randomisation

Consenting participants will be randomly assigned to either receive usual care or usual care combined with EMDR, utilising an internet-based system, following their CAPS-5 assessment. A researcher outside of the study team will undertake randomisation to ensure the CI remains blinded to study group allocation. Random allocation will occur in a 1:1 ratio, designating them to the control group (CG) for usual care or the intervention group (EMDR) for usual care plus EMDR.

#### Control group (CG)

Participants in the CG will receive the standard care package prescribed on hospital discharge, which may vary across study hospitals. Variations in standard care will be investigated through qualitative process evaluation and reported in the results manuscript. In case of adverse physical or psychological health conditions, they will access care through the usual available channels.

#### Intervention group (EMDR)

Participants randomised to the intervention group will receive the standard clinical care package following hospital discharge. Additionally, they will be referred to a participating adult NHS Psychological Therapies service using the established NHS–NHS referral system, identifying them as EMERALD participants. NHS Psychology teams will adhere to this research protocol for treatment. Any deviations from the protocol will be reported to the study team.

EMDR sessions, whether conducted via videoconference or face-to-face, will ideally commence within 4 weeks of referral and will be administered by trained EMDR therapists, who are supervised by a Consultant Clinical Psychologist. EMDR comprises eight phases, providing a structured treatment framework that supports consistency in session effects. The protocolised nature of EMDR facilitates training and replication in controlled studies. With participant and therapist agreement, some sessions will be recorded and assessed using the EMDR Fidelity Rating Scale (EFRS)^30^ to allow granular reporting of the delivered intervention. The EMDR protocol, reported according to the TIDieR (template for intervention description and replication) guidelines, is available in online supplemental file 2. Sessions will last up to 60 min, and therapist-recorded adherence will track the number of sessions offered versus those completed. Participants may receive up to 16 EMDR sessions based on the therapist’s ongoing assessment of need.

10.1136/bmjopen-2023-081969.supp2 Supplementary data

### Outcome measures

Primary outcome measures are feasibility and acceptability of trial process, to participants and staff.

Feasibility will be reported using the Consolidated Standards of Reporting Trials (CONSORT) statement as follows:

- Recruitment rate part A—we anticipate an average recruitment of 10 patients per month across the three participating sites. This is well above the median recruitment of 0.95 participants recruited per site per month, reported in a review of trials listed in the NIHR journals library (1997–2020).^31^
- Consent rate—number of patients recruited, expressed as a percentage of patients approached. Based on our previous work, we expect this to be greater than 30%.^28^
- Adherence will be determined by completion of ≥75% of planned EMDR sessions completed.
- Retention will be determined by ≥75% of participants completing the study follow-up assessment.

Acceptability will be determined by a qualitative process evaluation using semistructured interviews, and reported according to the Theoretical Framework of Acceptability.^32^ In addition, we will assess fidelity to the EMDR delivery model using the EFRS. This will enable us to account for variability in intervention delivery. Safety will be determined by assignment of causality of serious events. Events attributable to trial procedures will be reviewed by trial management board, study sponsor and the Research Ethics Committee (REC) to determine ongoing feasibility.

In addition to sociodemographic characteristics, and medical history (including ICU admission data), secondary outcome measures will be collected at baseline, 2 months and 12 months posthospital discharge to capture possible clinical outcomes, mediators, moderators and covariates that may be included in the subsequent, definitive effectiveness trial. A detailed description of each of these measures is provided in online supplemental file 3. All data will be stored securely, pseudonymised by study number, on the Qualtrics electronic database. The secondary outcome measures include the following:

10.1136/bmjopen-2023-081969.supp3 Supplementary data

- Change in PTSD symptom severity using the Impact of Events Scale-Revised (IES-R)^33^
- Change in categorical diagnosis of PTSD using IES-R.
- Post-traumatic stress score using Clinician administered PTSD scale for DSM-5 (CAPS-5)^34^
- Clinical Global Impression-Severity scale (CGI-S).^35^
- Sensitivity analysis: to determine whether PTSD symptom burden identified by IES-R corresponds with those identified by CAPS-5.
- Anxiety: Generalised Anxiety Disorder-7 (GAD-7)^36^
- Depression: Patient Health Questionnaire-9 (PHQ-9)^37^
- Quality of life EuroQol Five Dimension-Five level scale (EQ5D-5L).^38^
- Clinical Global Impression of Improvement (CGI-I).^35^

### Sample size

As this is a feasibility study, an a priori sample size calculation is not applicable. The findings will guide the sample size determination for a potential definitive RCT. Sample sizes of feasibility studies between 24 and 50 have been recommended to provide adequate estimate of SD for sample size calculation.^39 40^

To achieve this, a total of 160 patients will be enrolled in part A to assess feasibility adequately. Based on an expected incidence of 20%–25% post-ICU PTSD, we anticipate that around 40 patients will proceed to the part B RCT with an IES-R PTSD score≥22. The remaining 120 participants will continue in the observation arm, with a 12 month reassessment. Accounting for an estimated 25% mortality or loss to follow-up across all study arms, we anticipate approximately 30 participants completing the RCT and 90 participants completing the observation arm.

### Data plan and analysis

Recruitment, retention and trial completion data will be visually represented in a CONSORT diagram. Quantitative outcome analysis, encompassing measures such as IES-R, CAPS-5, PHQ-9, GAD-7 and EQ5D-5L, will primarily be descriptive, emphasising estimation. Baseline measures and outcomes will be summarised using appropriate descriptive statistics, complete with associated CIs. The focus of interpretation will centre on the implications of these results for the feasibility of the main trial. Furthermore, we will conduct a confirmatory factor analysis of the DSM-5’s four-factor PTSD diagnostic criteria, utilising data pooled from the CAPS-5 interviews.

### Qualitative process evaluation

Qualitative description will be employed to construct a comprehensive overview of participants’ and staff perceived experiences and the impact of the EMERALD study. This includes assessing the perceived burden associated with study participation and undertaking research activities. Qualitative interview data will serve to validate, elaborate on and broaden our understanding of the study’s acceptability and feasibility, while also shedding light on potential factors that may hinder or enhance the EMERALD study. This information will be invaluable in refining the design of the subsequent RCT.

### Method for obtaining and evaluating qualitative data

The process evaluation aligns with MRC guidance for complex intervention evaluations.^41^ To efficiently capture implementation processes, we will employ Rapid Assessment Procedure Informed Clinical Ethnography.^42^

*Stage 1: data collection* involves selecting a purposive, diverse sample of trial participants and psychological therapists, minimising bias by adapting the sample to study needs. Participants will be invited for recorded telephone or videoconference interviews at their convenience. We will use semistructured interviews guided by relevant objectives, incorporating patient and public involvement (PPI) recommendations, recent literature and a systematic review. See online supplemental material 4 for participant interview guide and psychological therapist interview guide. Sampling will continue until data saturation is reached, typically with 15–20 interviews.^43^ The questions will be open-ended, and we will take field notes while digitally recording and transcribing interviews. The data will be reviewed by a senior researcher within the team to assess the need for further data collection.

10.1136/bmjopen-2023-081969.supp4 Supplementary data

*Stage 2*: the anonymised data set will be securely stored and analysed using NVivo qualitative data software. The analysis will follow the theoretical framework of acceptability, deductively coding content into seven constructs;^32^ affective attitude, burden, intervention coherence, ethicality, opportunity costs, perceived effectiveness and self-efficacy.

Preliminary interpretation of emerging themes will be independently conducted, with consensus reached through discussion. Additional data collection will be considered if necessary. Agreed findings will be presented to a sample of study participants and PPI representatives to ensure validity and comprehensiveness.

*Stage 3*: will integrate qualitative findings with quantitative RCT data during the post-study interpretation phase. We will map data using a mixed-methods joint display,^44^ and providing a holistic understanding of predetermined study objectives following established principles.

### Safety considerations

Several systematic reviews have reported no adverse events attributable to EMDR. The intervention will be undertaken by suitably trained and experienced psychological therapists employed by the NHS. The service has an established and defined risk management and clinical governance structure. Online sessions will be compliant with Digital Approaches to therapy guidance from the British Psychological Society and NHS Digital. (This guidance contains expected standards relating to safeguarding, information governance, and GDPR.)

Participants who exhibit symptoms of intrusion/escalation will be treated according to the protocol unless it is determined that further treatment or escalation to emergency care may be necessary/indicated. If further treatment is required, the most appropriate course of action and referral pathway will be decided on a case-by-case basis by the psychology team. If deemed necessary, the CI will be unblinded to group allocation, to contribute to the safety discussion.

### Monitoring and trial oversight

Day-to-day management will be the joint responsibility of the CI, Senior Project Co-Ordinator and Co-Investigators. This project is part of a PhD study undertaken by Andrew Bates (CI) with supervision by the co-investigators and authors.

#### Monitoring

The CI will facilitate monitoring by the local quality manager, REC review and provide access to source data as required. Following any monitoring, a report will be provided which will summarise the visit and documents, along with any fi/ndings. The CI will be responsible for ensuring that all findings are addressed appropriately. The study group will review all events in a timely manner. Additional monitoring will be scheduled where there is evidence of suspicion of non-compliance with the study protocol.

### Patient and public involvement

PPI has shaped the study design, and this collaboration will persist throughout the project in the following ways:

#### Patient advisory group

An established PPI group attended advisory group meetings during project development. We are planning for meetings to occur every 6 months to review research findings, discuss key points, review press releases and dissemination outputs. Any study design amendments will be discussed and approved before submission.

#### Study management steering group

Two PPI members will serve as patient representatives in this decision-making group. They will oversee trial progress, review findings and outputs, approve project changes, and address arising issues, conflicts and risks in three meetings per year. One PPI group member will attend an intensive care conference to copresent study findings to clinical and academic leaders.

#### Patient groups and third sector

Study findings and dissemination outputs will be shared with and reviewed by patient groups and organisations such as ICU steps, EMDR UK, EMDR Europe and Anxiety UK. This ensures the inclusion of the patient perspective in the manuscript and keeps relevant stakeholders well informed.

Meetings will be conducted face-to-face with the option of videoconferencing for accessibility. A plain English research report, agenda and previous minutes will be circulated before each meeting, and meetings may be recorded with participant consent for later reference. Ongoing training tailored to individual needs will be provided for all participants, and the Public Involvement Lead for South Central Research Design Service will oversee ongoing PPI efforts.

## Ethics and dissemin§ation

This study obtained prior approval from the South Central -Hampshire A Research Ethics Committee (REC) (22/SC/0410) before approaching participants, who will also review protocol modifications. Ethics approval covers all NHS trial sites, which were activated before enrolling patients.

The trial will adhere to the principles outlined in the 18th World Medical Assembly’s recommendations from Helsinki 1964, as revised and recognised by governing laws and EU Directives. Consent to participate in the trial will be obtained only after providing a comprehensive explanation of treatment options, including conventional and widely accepted methods. The right of individuals to decline participation without specifying reasons will be respected.

Once a participant is enrolled in the trial, clinicians may administer alternative treatments beyond the protocol if they deem it in the participant’s best interest, with the reasons duly documented. The participant will continue within the trial for follow-up and data analysis based on their allocated treatment option. Likewise, participants are free to withdraw from protocol treatment and trial follow-up at any time without providing reasons, without affecting their subsequent treatment.

The CI will inform the REC on study completion. In cases of premature termination, the CI will promptly notify the REC, including the reasons for the early conclusion.

Within 1 year following the study’s conclusion, the CI will submit a final report containing results and any related publications or abstracts to the REC.

Dissemination activities will include but not be limited to:

- Publication in peer reviewed journals.
- Feedback to PPI study focus group.
- Feedback to study participants.
- Presentations to local clinical teams and managers and commissioners.
- Presentation at international conferences and within inter-disciplinary clinical networks.
- Public webinars, digital and social media.

## Discussion

The EMERALD study represents the second phase of our innovative exploration into whether EMDR can alleviate psychological distress after ICU discharge. Our mixed-methods approach, in line with MRC guidance for assessing complex healthcare interventions, enhances the study’s robustness.^41^ It allows us to capture cultural and contextual factors often missed in purely quantitative designs, thus improving the reliability of our findings and informing the design of our upcoming definitive RCT.

Building on the lessons from our prior study, CovEMERALD,^28^ we have incorporated screening for psychological distress before entry into the RCT, aligning with recent review recommendations.^45^ Adopting a 2 months posthospital discharge screening for PTSD follows both ICU rehabilitation^46^ and PTSD treatment guidelines.^24^ Furthermore, participants have the flexibility to choose either face-to-face or online intervention, without challenging participants’ physical or psychological vulnerabilities.

A noteworthy aspect of this project is the strong collaboration between clinical academics specialising in intensive care, psychiatry and psychology, bolstered by our patient representatives, individuals with valuable lived experiences.

It is important to interpret clinical findings from this study cautiously, as it is not powered to detect clinically significant differences between groups. Nevertheless, these outcomes will inform future power calculations for the definitive RCT.

## Supplementary Material

Reviewer comments

Author's manuscript
