# Supplementary material for: Mixed-methods randomised study exploring the feasibility and acceptability of eye-movement desensitisation and reprocessing for improving the mental health of traumatised survivors of intensive care following hospital discharge: protocol
Source: BMJ Open. 2024 Jan 29;14(1):e081969. doi: 10.1136/bmjopen-2023-081969 (PMC10826543; doi:10.1136/bmjopen-2023-081969)
Supplement: Supplementary data [file bmjopen-2023-081969supp002.pdf]

Supplementary file 2. EMDR treatment protocol reported according to TIDieR (Template for intervention description and replication)

Why: EMDR (Eye Movement Desensitization and Reprocessing) is hypothesised to alleviate post-traumatic symptoms among patients discharged from intensive care units (ICUs), by facilitating the adaptive processing of traumatic memories. The bilateral stimulation involved in EMDR is thought to assist in integrating memories of distressing experiences, potentially reducing the impact of trauma on mental health recovery. This study will investigate the feasibility and acceptability of delivering a randomised controlled trial (RCT) of EMDR following discharge from ICU.

What (material): No physical or informational materials were used during the intervention.

What (procedures): EMDR is a protocolised talking therapy which consists of 8 phases:

Phase 1: History taking and treatment planning: discuss participant history, with identification of traumatic events, develop a treatment plan, and assess participant’s internal and external resources.

Phase 2: **Preparation:** establish a therapeutic alliance through explanation of EMDR process, discuss expectations, concerns, and questions, and equip participant with techniques to address disturbance that may arise.

Phase 3: **Assessment:** identify a target event, including associated memories, feelings, and images. Ask the participant to rate the associated disturbance, from zero to ten, using the Subjective Units of Distress scale, (SUD) and the Validity of Cognition (VOC) scale.

Phase 4: **Desensitisation:** focussing on the target event, the participant will be asked to perform side-to-side eye movements, tapping or sounds. This phase will be repeated until SUD reduces to zero or one.

Phase 5: **Installation:** Once SUD has reduced to zero-one, the participant will be guided to associate a positive belief, with the target event, until it feels consistently true.

Phase 6: **Body scan:** the participant is guided to hold both the target event and positive belief in mind, while scanning their bodily sensations from head to toe. If they identify lingering disturbance, they will repeat phase 4, until reprocessing is complete.

Phases 7 and 8 are delivered at the end of each session and are designed to ensure safety.

Phase 7: Closure: The psychological therapist assists the participant to return to a state of calm.

Phase 8: Re-evaluation: The psychological therapist and EMDR (Eye-movement desensitisation and reprocessing)

participants discuss recently processed memories and identify future target memories and directions for treatment.

Who provided: EMDR was delivered by trained, experienced psychological therapists employed by the United Kingdom (UK) National Health Service (NHS). The therapists are undergoing monthly peer to peer support and are being supervised by an EMDR Europe accredited Consultant Clinical Psychologist.

How (mode of delivery; individual or group): EMDR is delivered face-to-face or via Internet teleconference, according to participant preference.

Where: Face-to-face sessions will take place within the NHS psychological therapies clinic. Online teleconference will take place via Microsoft Teams™. Where participants are unable to attend either face-to-face or Internet sessions then a tablet with Internet dongle will be provided by the study team.

When and how much: Sessions will be delivered weekly, last for up to 60 minutes, and are provided individually. Participants will receive up to 16 sessions of EMDR.

Tailoring: The nature of trauma focused psychological therapies necessitates a personalised approach to the intervention. However representative sample of sessions will be recorded and reviewed by an expert practitioner for fidelity using the EMDR Fidelity rating scale.

How well (planned): Adherence to EMDR intervention will be expressed as a percentage of sessions offered against sessions completed. Psychological therapists will complete a diary card which will be made available to the study team at the end of the intervention.
