# Supplementary material for: Mixed-methods randomised study exploring the feasibility and acceptability of eye-movement desensitisation and reprocessing for improving the mental health of traumatised survivors of intensive care following hospital discharge: protocol
Source: BMJ Open. 2024 Jan 29;14(1):e081969. doi: 10.1136/bmjopen-2023-081969 (PMC10826543; doi:10.1136/bmjopen-2023-081969)
Supplement: Supplementary data [file bmjopen-2023-081969supp003.pdf]

# Supplemental file 3. Secondary clinical outcome measures – description and timing

| | | Timepoint | | |
|---|---|---|---|---|
| **Measure** | **Description** | **Baseline** | **Two-months post-hospital discharge** | **12-months post-hospital discharge** |
| **Impact of Events Scale-Revised (IES-R)** (1) | IES-R is a 22-question patient reported outcome measure (PROM) widely used to assess symptoms of PTSD in critical care research(2,3), recommended by critical care core outcome dataset developers(4–6) and the International Conference of Harmonisation of Outcome Measures(7). The 22 questions cover symptoms of intrusion, avoidance, and hyperarousal. Participants indicate how distressing the symptoms have been over the last 7 days. Symptom severity can be 0 (not at all), 1 (a little bit), 2 (moderately), 3 (quite a bit), 4 (extremely), giving a total scoring range of 0 to 88.<br>A range of cut-offs for diagnosing PTSD have been identified in different populations. To maximise sensitivity, and minimise risk of leaving PTSD untreated, we will apply the lower cut-off of 22 and retrospectively conduct a sensitivity analysis against the CAPS-5. | X | X | X |
| **Clinician Administered PTSD Scale for DSM-5 (CAPS-5)**(8) | CAPS-5 is a structured diagnostic interview, considered the gold-standard assessment of PTSD symptoms. In addition to evaluating the 20 symptoms listed in the DSM-5, the questions focus on the onset and duration of these symptoms, the subjective distress experienced, how these symptoms impact an individual's social and occupational functioning, any improvement in symptoms since a prior CAPS assessment, the overall validity of the responses, the severity of PTSD as a whole, and the criteria for the dissociative subtype, which encompasses depersonalization and derealization.<br>CAPS-5 assessment will be conducted face-to-face or over the phone, (according to participant preference) methods which deliver comparable results. | | X (participants who have consented to part B only) | X (participants who have consented to part B only) |
| **Clinical Global Impression-Severity scale (CGI-S)**(9) | The CGI-S can be used to assess symptom severity and response to treatment. It requires a clinician to rate the severity of a patient's mental illness, on a seven-point scale ranging from; 1 – normal, not at all ill, 2 – borderline mentally ill, 3 – mildly ill, 4 – moderately ill, 5 – markedly ill, 6 – severely ill, 7 – among the most extremely ill patients. | | X (participants who have consented to part B only) | X (participants who have consented to part B only) |

| | | | | |
|---|---|---|---|---|
| **Clinical Global Impression-Improvement scale (CGI-I)**(9) | The CGI-I requires a clinician to assess degree of improvement since baseline, in a participant's symptoms, on a seven-point ranging from; 1-very much improved; 2-much improved; 3-minimally improved; 4-no change; 5-minimally worse; 6-much worse; 7-very much worse. | | X (participants who have consented to part B only) | X (participants who have consented to part B only) |
| **Patient Health Questionnaire (PHQ-9)**(10) | Self-administred, vaildated tool assesses depressive symptom severity(10–12). Scores are calculated by assigning 0 for 'not at all, 1- 'several days', 2 - 'more than half the days' or 3 – 'nearly every day' for responses to nine questions, giving a score in the range 0-27. PHQ-9 score of 0-4 demonstrates no - minimal depression severity. 5-9 = mild severity, 10-14 = moderate severity, 15-19 = moderately severe, 20-27 = severe. | X | X | X |
| **Generalised Anxiety Disorder-7 (GAD-7)**(13) | Seven-question, self-administered tool is validated to assess for anxiety symptom severity. Scores are calculated by assigning 0 for 'not at all, 1- 'several days', 2 - 'more than half the days' or 3 – 'nearly every day' for responses to nine questions, giving a score in the range 0-21. GAD-7 scores of 5, 10 and 15 represent cut-offs for mild, moderate and severe anxiety respectively. | X | X | X |
| **Health Related Quality of Life: Euroqual 5-level 5-Dimension (EQ-5D-5L)**(14) | Comprises five quality-of-life dimensions; mobility, self-care, usual activities, pain/discomfort and anxiety/depression. Participants report levels ranging from 'no problems' to 'extreme problems'. | X | X | X |
