# Supplementary material for: Mixed-methods randomised study exploring the feasibility and acceptability of eye-movement desensitisation and reprocessing for improving the mental health of traumatised survivors of intensive care following hospital discharge: protocol
Source: BMJ Open. 2024 Jan 29;14(1):e081969. doi: 10.1136/bmjopen-2023-081969 (PMC10826543; doi:10.1136/bmjopen-2023-081969)
Supplement: Supplementary data [file bmjopen-2023-081969supp004.pdf]

EMERALD – Participant Interview Guide – Intervention group

Introduction and orientation:

- Thank you for agreeing to take part in this research and for being interviewed today.
- Cover logistics of video conference interview and outline the plan if technology issues are experienced.
- Discuss recording and how we will store and use the information in this interview.
- The interview will cover a range of questions about your experiences. There are no right or wrong answers. We are very interested in your experience of the study as it will help us to design better studies in the future.
- If at any time you do not wish to answer a question or are unsure how to answer, that's okay.
- If you have any opinions that may seem challenging or critical, then that is okay too.

- Do you have any questions about this?
- Can I start the recording now?

Semi-structured interview questions:

**Introduction**	Can you talk me through how you became aware of the EMERALD study and run through your involvement?
**1. Affective Attitude:**<br><br>*How an individual feels about the intervention.*	I'd like you to think about how it felt taking part in the study.<br><br>• How did you feel towards EMDR? What informed that feeling?<br>• What did you like (or dislike) about EMDR? What were the best/worst parts?<br>• Enquire about feelings of calmness, positivity, discomfort, anxiety, feelings of panic etc; may need to probe for more information with appropriate reflective listening.
**2. Burden:**<br><br>*The amount of effort that was required to participate in the intervention*	I would like to discuss how much effort it took for you to undertake the EMERALD study – including any perceived difficulties or challenges?<br><br>• Did you experience any practical problems – online or face-to-face?<br>• Were there any consequences of receiving EMDR for you?<br>• What was the impact on your daily life?<br>• **Prompts:** may include cost, money, time commitment, or emotional burden<br>• If not yet addressed: What about other members of your household or family? (How did they support you if needed?)<br>• **Prompts:** help with internet access, use of technology, time, transport, financial
**3. Ethicality:**<br><br>*The extent to which the intervention has good fit*	I would like to explore the ethics of EMDR, such as respect, competence, responsibility, and integrity.<br><br>• Do you think there are any ethical issues with any aspect of taking part in the study?

with an individual's value system	• Can you describe any ethical implications to using EMDR in wider practice?<br>• Was there anything we could have done to make the study fairer?<br>• **Prompt:** In what ways do you think having EMDR fair or not fair?
4. **Intervention Coherence:**<br><br>*The extent to which the participant understands the intervention and how it works*	I notice that you attended XX sessions out of YY sessions arranged. I'd like to talk about your understanding of the EMDR.<br><br>• Having had EMDR, how do you think it helped or (doesn't/didn't help) regarding your symptoms of post-traumatic stress?<br>• How do you think it might work or not work?<br>• How much did you feel that EMDR was the right approach?<br>• What are your thoughts on the number of sessions? Do you think attending more (or fewer) sessions would change how effective it was?
5. **Opportunity Cost:**<br><br>*Experienced opportunity cost: The benefits, profits or values that were given up to engage in the intervention*	I'd like you to describe your feelings of the value and the potential costs of undertaking EMDR.<br><br>• What were the pros and cons of EMDR? Was there anything that you particularly liked or disliked?<br>• Was there anything that you had to give up so that you could have your EMDR?<br>• Do you have any reservations that you would like to discuss?
6. **Perceived Effectiveness:**<br><br>*The extent to which the intervention is perceived to have achieved its intended purpose.*	• How effective do/did you think (engaging with) EMDR was?<br>• How has EMDR affected the things that are important to you?<br>• **Prompts:** What weren't you able to do prior to EMDR that was important to you?<br>• Are you able to do this now? (work, home, social relationships)<br>• In what ways do you feel better/worse, emotionally, or physically?
7. **Self-efficacy:**<br><br>*The participant's confidence that they can perform the behaviour(s) required to participate in the intervention*	• How confident were you that you could (safely) take part in the study +/- the EMDR?<br>• How easy or difficult was it to stay engaged/concentrate for the whole session?<br>• **Prompt:** did it stir up any unpleasant or pleasant emotions?<br>• Do you think **you** had an ability to benefit?<br>• How did you address any challenges that we have previously discussed?

Question:
When considering all the things you've spoken about, what would be your overall summary of taking part in EMERALD?

Is there anything that you think could be done better?
Is there anything else you'd like to tell us?

Thank you for giving me your time again today and thank you for taking part in our study.

EMERALD – Psychological therapist Interview Guide

Introduction and orientation

Semi-structured interview questions:

**Introduction**	Can you talk me through how you became aware of the EMERALD study and run through your involvement?<br>I'd like you to consider the study group meetings, referrals, and delivery of the EMDR.
1. **Affective Attitude:**<br><br>*How an individual feels about the intervention.*	How did it feel to be taking part in the study.<br><br>**Prompts:**<br>Emotionally, did you enjoy it?<br><br>Enquire about anything that you found surprising, uncomfortable, or anxiety provoking; may need to probe for more information with appropriate reflective listening.
2. **Burden:**<br><br>*The amount of effort that was required to participate in the intervention*	I would like to discuss how much effort you feel it took to undertake the EMERALD study – you perception – any difficulties or challenges?<br><br>Did you experience any practical problems –online or face-to-face, burden of the additional workload?<br><br>What was the impact on your working life – time commitment and emotional strain.<br><br>**Prompt:** could include cost, money, time/workload, or emotional burden<br><br>Did you experience any (other) burden(s) because of your involvement?<br><br>**Prompt:** help with internet access, financial
3. **Ethicality:**<br><br>*The extent to which the intervention has good fit with an individual's value system*	Do you think there are any ethical issues with any aspect of the study?<br><br>What about the randomisation, do you think there are ethical issues some people getting or not getting EMDR when traumatised?<br><br>**Prompt:** In what ways do you think having EMDR or not having EMDR is fair or not fair?<br><br>Was there anything we could have done to make the study fairer?

4. **Intervention Coherence:**<br><br>*The extent to which the participant understands the intervention and how it works*	What is your understanding of EMDR and how it may be applicable with these participants?<br><br>What do think was the aim of the EMDR?<br><br>Did it seem sensible to use for post-ICU traumatic stress?<br><br>How might it work for these patients?<br><br>Do you think attending more (or fewer) sessions would change how effective it was?
5. **Opportunity Cost:**<br><br>*Experienced opportunity cost: The benefits, profits or values that were given up to engage in the intervention*	Could you describe your feelings of the value of undertaking EMDR?<br><br>**Prompt:** do you think this was better or worse than alternatives, including the option of doing nothing?<br><br>Do you have any reservations that you would like to discuss?
6. **Perceived Effectiveness:**<br><br>*The extent to which the intervention is perceived to have achieved its intended purpose.*	Do you think that EMDR has been effective for your participants?<br><br>**Prompt:** How do you feel it may have affected various aspects of their life? (work, home, social relationships)<br><br>Do you think they feel better, emotionally, or physically?
7. **Self-efficacy:**<br><br>*The participant's confidence that they can perform the behaviour(s) required to participate in the intervention*	How confident were you that you could deliver the study/EMDR as per the protocol?<br><br>**Prompt:** did it stir up any unpleasant emotions?<br><br>Do you think **you** were able to benefit?<br><br>How did you address any challenges that we have previously discussed?

We are very near to the end of our interview today and I would like to hear about how you felt overall.

Question:
When considering all the things you've spoken about, what would be your overall summary of taking part in EMERALD?

Is there anything that you think could be done better?

Is there anything else you'd like to tell us?

Thank you for giving me your time again today and thank you for taking part in our study.
