# Supplementary material for: Mixed-methods randomised study exploring the feasibility and acceptability of eye-movement desensitisation and reprocessing for improving the mental health of traumatised survivors of intensive care following hospital discharge: protocol
Source: BMJ Open. 2024 Jan 29;14(1):e081969. doi: 10.1136/bmjopen-2023-081969 (PMC10826543; doi:10.1136/bmjopen-2023-081969)
Supplement: Author's manuscript [file bmjopen-2023-081969.draft_revisions.pdf]

BMJ Open

BMJ Open is committed to open peer review. As part of this commitment we make the peer review history of every article we publish publicly available.

When an article is published we post the peer reviewers' comments and the authors' responses online. We also post the versions of the paper that were used during peer review. These are the versions that the peer review comments apply to.

The versions of the paper that follow are the versions that were submitted during the peer review process. They are not the versions of record or the final published versions. They should not be cited or distributed as the published version of this manuscript.

BMJ Open is an open access journal and the full, final, typeset and author-corrected version of record of the manuscript is available on our site with no access controls, subscription charges or pay-per-view fees (http://bmjopen.bmj.com).

If you have any questions on BMJ Open's open peer review process please email info.bmjopen@bmj.com

BMJ Open

Exploring the Feasibility and Acceptability of Eye-Movement Desensitisation and Reprocessing for post-intensive care mental health: A Mixed Methods Randomised Study protocol

Journal:	*BMJ Open*
Manuscript ID	bmjopen-2023-081969
Article Type:	Protocol
Date Submitted by the Author:	10-Nov-2023

Complete List of Authors:	Bates, Andrew; University Hospital Southampton NHS Foundation Trust, Perioperative and Critical Care Theme, NIHR Southampton Biomedical Research Centre; University of Southampton, Clinical and Experimental Sciences, Faculty of Medicine<br>Golding, Hannah; University Hospital Southampton NHS Foundation Trust, Perioperative and Critical Care Theme, NIHR Southampton Biomedical Research Centre<br>Rushbrook, Sophie; Dorset HealthCare University NHS Foundation Trust<br>Highfield, Julie; Cardiff and Vale University Health Board<br>Pattison, Natalie; University of Hertfordshire; East and North Hertfordshire NHS Trust<br>Baldwin, David ; University of Southampton Faculty of Medicine, Clinical and Experimental Sciences; Southern Health NHS Foundation Trust<br>Grocott, Michael P. W.; University Hospital Southampton NHS Foundation Trust, Perioperative and Critical Care Theme, NIHR Southampton Biomedical Research Centre; University of Southampton Faculty of Medicine, Clinical and Experimental Sciences<br>Cusack, Rebecca; Southampton University Hospitals NHS Trust, Perioperative and Critical Care Theme, NIHR Southampton Biomedical Research Centre; University of Southampton Faculty of Medicine

Keywords:	PSYCHIATRY, Anxiety disorders < PSYCHIATRY, INTENSIVE & CRITICAL CARE

SCHOLARONE™
Manuscripts

Title

Exploring the Feasibility and Acceptability of Eye-Movement Desensitisation and Reprocessing for post-intensive care mental health: A Mixed Methods Randomised Study

Authors

Andrew Bates[1,2], Hannah Golding[2], Sophie Rushbrook[3], Julie Highfield[4], Natalie Pattison[5,6], David S. Baldwin[1,7,8], Michael P W Grocott[1,2], and Rebecca Cusack[1,2]

Corresponding author: Andrew Bates; a.bates@soton.ac.uk

For peer review only

Abstract

Introduction

Post-traumatic symptoms are common among patients discharged from intensive care units (ICUs), adversely affecting well-being, increasing healthcare utilisation, and delaying return to work. Non-pharmacological approaches (e.g., music, therapeutic touch, patient diaries) have been suggested as candidate interventions and trauma-focused psychological interventions have been endorsed by international bodies. Neither category of intervention is supported by evidence of clinical effectiveness in patients who have been critically ill. This study assesses the feasibility and acceptability of using eye-movement desensitization and reprocessing (EMDR) to improve the mental health of ICU survivors.

Methods and analysis

EMERALD is a multicentre, two-part consent, pilot feasibility study, recruiting discharged ICU survivors from three hospitals in the United Kingdom. We are gathering demographics and measuring post-traumatic symptoms, anxiety, depression, and quality of life at baseline. Two months after discharge, participants are screened for symptoms of post-traumatic stress disorder (PTSD) using the Impact of Events Scale-Revised (IES-R). Patients with IES-R scores <22 continue in an observation arm for 12-month follow-up. IES-R scores ≥22 indicate above-threshold PTSD symptoms and trigger invitation to consent for Part B: a randomised controlled trial (RCT) of EMDR vs. usual care, with 1:1 randomisation. The study assesses feasibility (recruitment, retention, intervention fidelity) and acceptability (through semi-structured interviews), using a theoretical acceptability framework. Clinical outcomes (PTSD, anxiety, depression, and quality of life) are collected at baseline, two- and 12-months, informing power calculations for a definitive RCT, with quantitative and qualitative data convergence guiding RCT refinements.

Ethics and dissemination

This study has undergone external expert peer review and is funded by a National Institute for Health and Care Research Clinical Doctoral Research Fellowship (NIHR CDRF) awarded to Andrew Bates (NIHR302160). Ethical approval has been granted by South Central – Hampshire A Research Ethics Committee (IRAS number: 317291) and the study is registered on ClinicalTrials.gov: NCT05591625.
Results will be disseminated through the lay media, social media, peer reviewed publication and conference presentation.

Keywords

Critical care; Eye Movement Desensitization and Reprocessing; stress disorder, post-traumatic; anxiety

Article summary

Strengths and limitations of this study

- First study to systematically evaluate the impact of EMDR on the mental health of adult ICU survivors.
- Adheres to Medical Research Council guidance for evaluating complex healthcare interventions.
- Mixed methods probe feasibility and acceptability enabling us to address cultural and contextual factors.
- Consistent with existing clinical pathways and best practice guidance.
- Not powered to detect between-group, clinically significant differences in post-traumatic symptoms.
- Findings will inform the design of a subsequent, fully powered RCT.

Introduction

Background and rationale

Critically ill patients in intensive care units (ICUs) receive life-saving treatment, yet the burden of long-term physical, cognitive, and mental health issues, collectively known as 'post-intensive care syndrome', is significant(1). Global ICU admissions are on the rise(2) and there is growing recognition of the need to address post-ICU survivorship as a defining challenge in 21st-century intensive care medicine(3). Despite this, healthcare providers often overlook this phase(4), resulting in multiple care transitions away from clinicians with an understanding of the underlying aetiology(5).

Amidst the existential threat of critical illness, patients endure invasive treatments, potent psychoactive drugs, a busy and confusing environment, and limited communication, leading to normal acute anxiety responses(6). However, a substantial proportion continue to suffer unpleasant psychological and somatic symptoms. Post-ICU discharge, 20-25% experience

For peer review only - http://bmjopen.bmj.com/site/about/guidelines.xhtml

symptoms similar to those of post-traumatic stress disorder (PTSD) (7), with over 30% and 40% experiencing depression(8) and anxiety(9), respectively. These symptoms can be persistent(10), co-occurring(11), and are associated with adverse outcomes including reduced quality of life, increased healthcare utilisation and delayed return to work(8,11,12).

Despite this, access to clinical psychology remains underrepresented in United Kingdom (UK) ICU recovery services(14). Interventions like music therapy(15), therapeutic touch(16), and patient diaries(17) have been explored, but systematic reviews reveal that definitive evidence of long-term effect is lacking. Trauma-focused psychological therapies, such as Eye Movement Desensitization and Reprocessing (EMDR), offer some promise, with meta-analyses showing significant reductions in PTSD, anxiety, and depression for treating a diverse range of traumatised populations(18,19). EMDR is cost-effective(20) and is internationally recommended by major organisations for trauma-related symptoms(21–24).

Recent investigations of EMDR's effectiveness in treating medical event-induced trauma, following cancer, stroke, cardiac events, and multiple sclerosis have yielded promising but inconclusive findings(25). Case studies with ICU survivors(26,27) and our own novel work with survivors of COVID-19 related critical illness(28) also show promise, underscoring the need for systematic evaluation in this population. However, definitive evidence of benefit is not available.

Objectives:

The primary objective of the EMERALD study is to evaluate the feasibility and acceptability of an EMDR intervention for adult patients displaying traumatic stress symptoms following ICU discharge. These findings will guide the design of a robust, fully powered randomised controlled trial (RCT), aligning with Medical Research Council (MRC) guidance on evaluating complex medical interventions. Secondary clinical outcomes will inform the selection of a primary outcome for the larger trial and provide variance estimates for sample size calculations. Additionally, a light-touch observation arm will offer insights into the mental health trajectory of ICU survivors without traumatic stress symptoms two-months after hospital discharge.

Methods: Participants, interventions, and outcomes

Design

This is a multicentre, mixed methods, randomised controlled pilot feasibility study, with a two-part consent process and is reported using the SPIRIT reporting guidelines(29). Initially, all participants enter Part A, which is an observational study, where they complete a series of mental health questionnaires at baseline, two-months, and 12-months post-hospital discharge. If a participant shows symptoms of post-traumatic stress at the two-month mark (scoring ≥22 on the Impact Events Scale-Revised (IES-R)), they are invited to consider participating in Part B, which is an interventional study of EMDR vs. standard care. Those

For peer review only - http://bmjopen.bmj.com/site/about/guidelines.xhtml

without post-traumatic stress symptoms at 2 months (≤21 on the IES-R) or those who decline participation in Part B will be offered continuation of the observation arm. All participants from both Part A and Part B repeat the study assessments at 12 months post-hospital discharge. See Figure 1 for the participant timeline.

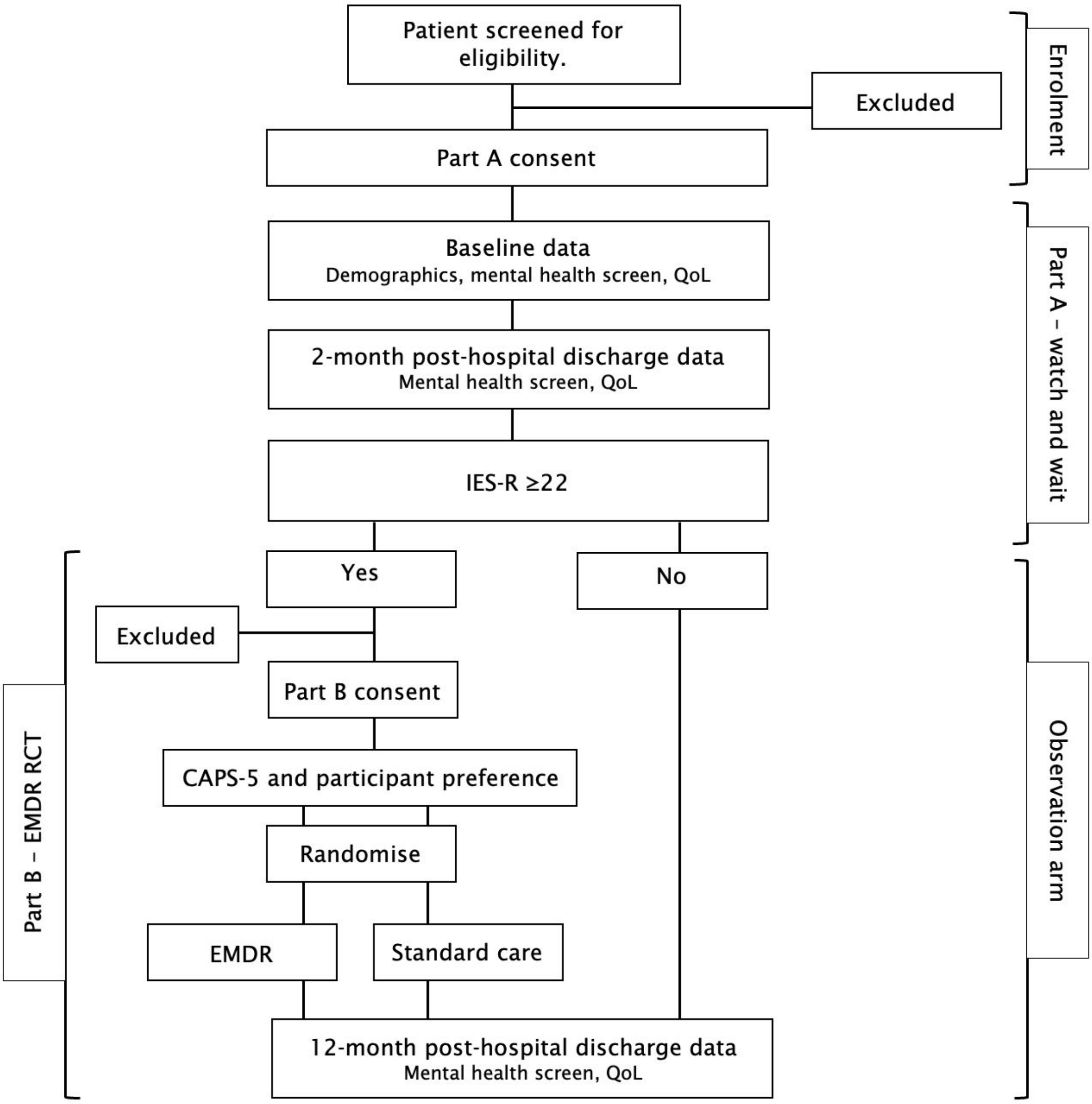

Figure 1. **EMERALD participant timeline.** IES-R, Impact of Events Scale-Revised; QoL, Quality of Life; RCT, Randomised Controlled Trial; EMDR, Eye-Movement Desensitisation and Reprocessing; CAPS-5, Clinician Administered PTSD Scale for DSM-5

For peer review only - http://bmjopen.bmj.com/site/about/guidelines.xhtml

Study setting

The study is sponsored by the University Hospital Southampton National Health Service (NHS) Foundation Trust (FT). Recruitment will occur after adult patients are discharged from three adult NHS ICUs in the UK: University Hospital Southampton, Royal Bournemouth Hospital, and Poole General Hospital. The intervention will be provided through NHS psychological therapy services in proximity to the study participants, specifically Southern Health NHS FT and Dorset Healthcare University NHS FT.

Part A participant recruitment

Recruitment is anticipated to occur between February 2023 and May 2024. Eligibility screening will target consecutive patients discharged from the participating ICUs. Research staff will approach eligible patients on hospital wards or within two months following hospital discharge, via a telephone call or email, providing a participant information sheet. Patients will be invited to complete an informed consent form (ICF), accessible electronically through Qualtrics™ on tablet devices provided by the trial team, via an emailed link or on paper to suit patient preference. This initial consent pertains to their participation in the observational study (Part A), involving baseline data collection and psychometric assessments, with a follow-up evaluation at two months and 12-months following hospital discharge.

Eligibility criteria

Eligibility will be determined by hospital research nurses acting under delegated authority of the local Principal Investigator. Patients will be eligible for part A if they meet the following criteria:

- Survivor of an intensive care admission, who received level 3 care for >24 hours.
- Aged ≥18 years.
- Capacity to provide informed consent.

Patients will be excluded if they meet any of the following criteria:

- Pre-existing cognitive impairment such as dementia.
- Pre-existing diagnosis of psychosis.
- Not expected to survive beyond hospital discharge.
- Traumatic brain injury.

Baseline data collection

Research staff will collect demographic data, medical history, and ICU admission history following consent. All participants will complete the Impact of Events Scale-Revised (IES-R), Patient Health Questionnaire-9, (PHQ-9) Generalised Anxiety Disorder 7, (GAD-7) and the Euroqol 5 Dimension 5 Level. (EQ-5D-5L)

For peer review only

	Baseline	2 months post-discharge	3-9 months post-discharge	12-months post-discharge
**Informed Consent**	X<br>Part A	X*<br>Part B		
**Demographics**	X			
**IES-R**	X	X		X
**CAPS-5, CGI-S***		X*		X*
**PHQ-9**	X	X		X
**GAD-7**	X	X		X
**EQ-5D-5L**	X	X		X

Two-month post-hospital discharge assessment

All participants will be requested to repeat the IES-R, PHQ-9, GAD-7, and EQ-5D-5L. These patient-reported outcome measures can be completed electronically via an emailed link or by using paper versions sent with a prepaid return envelope.

The study team will review the IES-R responses. Participants with a total score ≥22, indicative of post-traumatic stress symptoms, will be approached to consider participation in an EMDR vs. usual care RCT (Part B).

Participants without symptoms (IES-R≤21) or those not interested or unable to participate in the RCT will continue in the observational study, completing the 12-month follow-up assessment.

12-month follow-up assessment

Research staff will ask all participants, in both the observation group (Part A only) and RCT (Part A and Part B), to repeat the IES-R, PHQ-9, GAD-7 and the EQ-5D-5L, at 12-months post-hospital discharge. See Table 1 for the full study schedule of events.

For peer review only

**EMDR Intervention**			X*	
**IES-R, PHQ-9, GAD-7 (EMDR group only)**				
**Randomisation preference***		X*		
**Process Evaluation**			X	X

Table 1: EMERALD study schedule of events. X* for participants consenting to part B of the study only.

For peer review only

Part B participant recruitment

Participants scoring ≥22 on the 2-month IES-R will receive a phone call or email from the study team, inviting them to consider consenting to Part B, the EMDR vs. usual care RCT. The Part B PIS and ICF will be accessible electronically or via postal delivery. Those who consent to Part B will first undergo a Clinician Administered PTSD Scale for Diagnostic and Statistical Manual of Mental Disorders, fifth edition (CAPS-5) assessment to evaluate PTSD symptoms and a Clinical Global Impression of Illness Severity (CGI-S) assessment with the Chief Investigator (CI). Additionally, participants will be asked to rate their preference for study arm strength using a Likert scale ranging from 0 to 10.

Randomisation

Consenting participants will be randomly assigned to either receive usual care or usual care combined with EMDR, utilising an internet-based system, following their CAPS-5 assessment. A researcher outside of the study team will undertake randomisation, to ensure the CI remains blinded to study group allocation. Random allocation will occur in a 1:1 ratio, designating them to the control group (CG) for usual care or the intervention group (EMDR) for usual care plus EMDR.

Control Group (CG): Participants in the control group will receive the standard care package prescribed upon hospital discharge, which may vary across study hospitals. Variations in standard care will be investigated through qualitative process evaluation and reported in the results manuscript. In case of adverse physical or psychological health conditions, they will access care through the usual available channels.

Intervention Group (EMDR): Participants randomised to the intervention group will receive the standard clinical care package following hospital discharge. Additionally, they will be referred to a participating adult NHS Psychological Therapies service using the established NHS-NHS referral system, identifying them as EMERALD participants. NHS Psychology teams will adhere to this research protocol for treatment. Any deviations from the protocol will be reported to the study team.

EMDR sessions, whether conducted via videoconference or face-to-face, will ideally commence within 4 weeks of referral and will be administered by trained EMDR therapists, who are supervised by a Consultant Clinical Psychologist. EMDR comprises eight phases, providing a structured treatment framework that supports consistency in session effects. The protocolised nature of EMDR facilitates training and replication in controlled studies. With participant and therapist agreement, some sessions will be recorded and assessed using the EMDR Fidelity Rating Scale (EFRS)(30) to allow granular reporting of the delivered intervention. An outline of the EMDR protocol, reported according to the TIDieR (Template for intervention description and replication) guidelines, is available in supplementary material (appendix 1). Sessions will last up to 60 minutes, and therapist-recorded adherence will track the number of sessions offered versus those completed. Participants may receive up to 16 EMDR sessions, based on the therapist's ongoing assessment of need.

Outcome measures

Primary outcome measures are feasibility and acceptability of trial process, to participants and staff.

Feasibility will be reported using the CONSORT statement as:

- Recruitment rate part A – we anticipate an average recruitment of 10 patients per month across the three participating sites. This is well above the median recruitment of 0.95 participants recruited per site per month, reported in a review of trials listed in the NIHR journals library (1997-2020)(31).
- Consent rate – number of patients recruited, expressed as a percentage of patients approached. Based on our previous work we expect this to be greater than 30%(28).
- Adherence will be determined by completion of ≥75% of planned EMDR sessions completed.
- Retention will be determined by ≥75% of participants completing the study follow-up assessment.

Acceptability will be determined by a qualitative process evaluation using semi-structured interviews, and reported according to the Theoretical Framework of Acceptability(32).

In addition to sociodemographic characteristics, and medical history (including ICU admission data) secondary outcome measures will be collected at baseline, 2-months, and 12-months post-hospital discharge to capture possible clinical outcomes, mediators, moderators, and covariates that may be included in the subsequent, definitive effectiveness

For peer review only - http://bmjopen.bmj.com/site/about/guidelines.xhtml

trial. A detailed description of each of these measures is provided in supplementary material appendix 2. All data will be stored securely, pseudonymised by study number, on the Qualtrics^{TM} electronic database. The clinical outcome measures include:

- Change in PTSD symptom severity using the Impact of Events Scale – Revised (IES-R)(33)
- Change in categorical diagnosis of PTSD using IES-R.
- Post-traumatic stress score using Clinician administered PTSD scale for DSM-5 (CAPS-5)(34)
- Clinical Global Impression-Severity scale (CGI-S)(35)
- Anxiety: Generalised Anxiety Disorder-7 (GAD-7)(36)
- Depression: Patient Health Questionnaire-9 (PHQ-9)(37)
- Quality of life EuroQol Five Dimension- Five level scale (EQ5D -5L)(38)
- Clinical Global Impression of Improvement (CGI-I)(35)

Sample size

As this is a feasibility study, an *a priori* sample size calculation is not applicable. The findings will guide the sample size determination for a potential definitive RCT. Sample sizes of feasibility studies between 24 and 50 have been recommended, to provide adequate estimate of standard deviation for sample size calculation(39,40).
To achieve this, a total of 160 patients will be enrolled in Part A to assess feasibility adequately. Based on an expected incidence of 20-25% post-ICU PTSD, we anticipate that around 40 patients will proceed to the Part B RCT with an IES-R PTSD score ≥22. The remaining 120 participants will continue in the observation arm, with a 12-month re-assessment. Accounting for an estimated 25% mortality or loss to follow-up across all study arms, we anticipate approximately 30 participants completing the RCT and 90 participants completing the observation arm.

Data plan and analysis

Recruitment, retention, and trial completion data will be visually represented in a CONSORT diagram. Quantitative outcome analysis, encompassing measures such as IES-R, CAPS-5, PHQ-9, GAD-7, and EQ5D-5L, will primarily be descriptive, emphasizing estimation. Baseline measures and outcomes will be summarised using appropriate descriptive statistics, complete with associated confidence intervals. The focus of interpretation will centre on the implications of these results for the feasibility of the main trial. Furthermore, we will conduct a confirmatory factor analysis (CFA) of the DSM-5's four-factor PTSD diagnostic criteria, utilising data pooled from the CAPS-5 interviews.

Qualitative process evaluation

Qualitative description will be employed to construct a comprehensive overview of participants' and staff perceived experiences and the impact of the EMERALD study. This

For peer review only

includes assessing the perceived burden associated with study participation and undertaking research activities. Qualitative interview data will serve to validate, elaborate upon, and broaden our understanding of the study's acceptability and feasibility, while also shedding light on potential factors that may hinder or enhance the EMERALD study. This information will be invaluable in refining the design of the subsequent RCT.

Method for obtaining and evaluating qualitative data.

The process evaluation aligns with MRC guidance for complex intervention evaluations(41). However, this guidance has faced criticism for its lack of theory-driven approaches(42), potentially leading to limited insight into contextual factors and mechanisms of change(43). To efficiently capture implementation processes, we will employ Rapid Assessment Procedure Informed Clinical Ethnography(44).

Stage 1- data collection involves selecting a purposive, diverse sample of trial participants and psychological therapists, minimizing bias by adapting the sample to study needs. Participants will be invited for recorded telephone or videoconference interviews at their convenience. We will use semi-structured interviews guided by relevant objectives, incorporating patient and public involvement (PPI) recommendations, recent literature, and a systematic review. Sampling will continue until data saturation is reached, typically with 15-20 interviews(45). The questions will be open-ended, and we will take field notes while digitally recording and transcribing interviews. The data will be reviewed by a senior researcher within the team, to assess the need for further data collection.

Stage 2 - the anonymised dataset will be securely stored and analysed using NVivo™ qualitative data software. The analysis will follow the theoretical framework of acceptability, deductively coding content into seven constructs(32); affective attitude, burden, intervention coherence, ethicality, opportunity costs, perceived effectiveness, and self-efficacy.

Preliminary interpretation of emerging themes will be independently conducted, with consensus reached through discussion. Additional data collection will be considered if necessary. Agreed findings will be presented to a sample of study participants and PPI representatives to ensure validity and comprehensiveness.

Stage 3 - will integrate qualitative findings with quantitative RCT data during the post-study interpretation phase. We will map data using a mixed methods joint display(46), and providing a holistic understanding of predetermined study objectives following established principles.

For peer review only - http://bmjopen.bmj.com/site/about/guidelines.xhtml

Safety considerations

Several systematic reviews have reported no adverse events attributable to EMDR. The intervention will be undertaken by suitably trained and experienced psychological therapists employed by the NHS. The service has an established and defined risk management and clinical governance structure. Online sessions will be compliant with Digital Approaches to therapy guidance from the British Psychological Society and NHS Digital. (This guidance contains expected standards relating to safeguarding, information governance, and GDPR).

Participants who exhibit symptoms of intrusion/ escalation will be treated according to the protocol unless it is determined that further treatment or escalation to emergency care may be necessary/ indicated. If further treatment is required, the most appropriate course of action and referral pathway will be decided on a case-by-case basis by the psychology team. If deemed necessary the Chief Investigator will be unblinded to group allocation, to contribute to the safety discussion.

Monitoring and trial oversight

Day to day management will be the joint responsibility of the Chief Investigator, Senior Project Co- Ordinator and Co-Investigators. This project is part of a PhD study undertaken by Andrew Bates (CI) with supervision by the co-investigators and authors.

Monitoring: The CI will facilitate monitoring by the local quality manager, REC review and provide access to source data as required. Following any monitoring a report will be provided which will summarise the visit and documents, along with any findings. The CI will be responsible for ensuring that all findings are addressed appropriately. The study group will review all events in a timely manner. Additional monitoring will be scheduled where there is evidence of suspicion of non-compliance with the study protocol.

Patient and public involvement

Patient and public involvement (PPI) has shaped the study design, and this collaboration will persist throughout the project in the following ways:

Patient Advisory Group: An established PPI group attended advisory group meetings during project development. We are planning for meetings to occur every six months to review research findings, discuss key points, review press releases and dissemination outputs. Any study design amendments will be discussed and approved before submission.

Study Management Steering Group: Two PPI members will serve as patient representatives in this decision-making group. They will oversee trial progress, review findings and outputs, approve project changes, and address arising issues, conflicts, and risks in three meetings per year. One PPI group member will attend an Intensive Care conference to co-present study findings to clinical and academic leaders.

For peer review only - http://bmjopen.bmj.com/site/about/guidelines.xhtml

Patient Groups and Third Sector: Study findings and dissemination outputs will be shared with and reviewed by patient groups and organisations such as ICU Steps, EMDR UK, EMDR Europe, and Anxiety UK. This ensures the inclusion of the patient perspective in the manuscript and keeps relevant stakeholders well-informed.

Meetings will be conducted face-to-face with the option of videoconferencing for accessibility. A plain English research report, agenda, and previous minutes will be circulated before each meeting, and meetings may be recorded with participant consent for later reference. Ongoing training tailored to individual needs will be provided for all participants, and the Public Involvement Lead for South Central Research Design Service will oversee ongoing PPI efforts.

Ethics and dissemination

This study obtained prior approval from the South Central - Hampshire A Research Ethics Committee (REC) (22/SC/0410) before approaching participants, who will also review protocol modifications. Each trial site was activated before enrolling patients.

The trial will adhere to the principles outlined in the 18th World Medical Assembly's recommendations from Helsinki 1964, as revised and recognized by governing laws and EU Directives. Consent to participate in the trial will be obtained only after providing a comprehensive explanation of treatment options, including conventional and widely accepted methods. The right of individuals to decline participation without specifying reasons will be respected.

Once a participant is enrolled in the trial, clinicians may administer alternative treatments beyond the protocol if they deem it in the participant's best interest, with the reasons duly documented. The participant will continue within the trial for follow-up and data analysis based on their allocated treatment option. Likewise, participants are free to withdraw from protocol treatment and trial follow-up at any time without providing reasons, without affecting their subsequent treatment.

The Chief Investigator will inform the REC upon study completion. In cases of premature termination, the CI will promptly notify the REC, including the reasons for the early conclusion.

Within one year following the study's conclusion, the CI will submit a final report containing results and any related publications or abstracts to the REC.

Dissemination activities will include but not be limited to:

- Publication in peer reviewed journals.
- Feedback to PPI study focus group.
- Feedback to study participants.
- Presentations to local clinical teams and managers and commissioners.

For peer review only - http://bmjopen.bmj.com/site/about/guidelines.xhtml

- Presentation at international conferences and within inter-disciplinary clinical networks.
- Public webinars, digital and social media.

Discussion

The EMERALD study represents the second phase of our innovative exploration into whether EMDR can alleviate psychological distress after ICU discharge. Our mixed methods approach, in line with MRC guidance for assessing complex healthcare interventions, enhances the study's robustness(41). It allows us to capture cultural and contextual factors often missed in purely quantitative designs, thus improving the reliability of our findings, and informing the design of our upcoming definitive RCT.

Building on the lessons from our prior study, CovEMERALD(28), we have incorporated screening for psychological distress before entry into the RCT, aligning with recent review recommendations(47). Adopting a two-months post-hospital discharge screening for PTSD follows both ICU rehabilitation(48) and PTSD treatment guidelines(24). Furthermore, participants have the flexibility to choose either face-to-face or online intervention, without challenging participants' physical or psychological vulnerabilities.

A noteworthy aspect of this project is the strong collaboration between clinical academics specialising in intensive care, psychiatry, and psychology, bolstered by our patient representatives, individuals with valuable lived experiences.

It is important to interpret clinical findings from this study cautiously, as it is not powered to detect clinically significant differences between groups. Nevertheless, these outcomes will inform future power calculations for the definitive RCT.

Author affiliations

[1]Clinical and Experimental Sciences, Faculty of Medicine, University of Southampton, UK
[2] Perioperative and Critical Care Theme, NIHR Southampton Biomedical Research Centre, University Hospital Southampton / University of Southampton, Southampton, UK
[3]Dorset Healthcare University National Health Service Foundation Trust
[4]Cardiff and Vale University Health Board, UK
[5]University of Hertfordshire
[6]East and North Hertfordshire NHS Trust
[7]Southern Health NHS Foundation Trust
[8]University Department of Psychiatry and Mental Health, University of Cape Town, South Africa

Twitter

Andrew Bates @AndyCBates and Michael Grocott @mike_grocott

For peer review only

ORCID iDs

Andrew Bates https://orcid.org/0000-0002-3614-0270
Michael Grocott https://orcid.org/0000-0002-9484-7581

Acknowledgements

We acknowledge the contribution of our public and patient involvement (PPI) team. Thank you to the NHS intervention delivery teams for helping to design and deliver the EMDR; Janet Hathaway, Tara Walker, Shalini Ramani, Vanessa Ballard, Emer Young, Michelle Gormley, and Rachel Sadler. Thanks to the research nurses at recruiting sites; Jonathan Biss, Karen Salmon, Sally Pitts, Debbie Branney, Yasmin De'Ath, and Emma Langridge. Thanks also to the nurses leading the ICU follow-up services who understand and appreciate the complex convalescence faced by our patients; Fiona Hall, Rachael Hopkins, and Gemma Turnbull. AB would like to thank Dr Kay Mitchell, Professor Robert Crouch, the team at Southampton Academy of Research, Research Support Service South Central and University Hospital Southampton Research and Development for their support with the NIHR Clinical Doctoral Research Fellowship application and ongoing studies.

Funding

This work was funded by an NIHR Clinical Doctoral Research Fellowship awarded to AB. The views expressed are those of the authors and not necessarily those of the National Health Service the National Institute for Health and Social Care Research or the Department of Health.

Competing interests

None declared.

Provenance and peer review

This work has undergone expert external peer review by the NIHR Clinical Doctoral Research Fellowship panel. It has undergone expert internal peer review by study sponsors at University Hospital Southampton.

Authors contributions

AB and SR conceived the research idea. AB developed the theory, research plan, drafted the manuscript and is Chief Investigator for the study. HG acted as project manager during study set-up. All authors (AB, HG, SR, JH, NP, DSB, MG, and RC) contributed to the study development and have reviewed, revised, and approved this manuscript.

Word count: 3443

For peer review only

References

1. Desai S V., Law TJ, Needham DM. Long-term complications of critical care. Crit Care Med. 2011 Feb;39(2):371–9.

2. Adhikari NKJ, Fowler RA, Bhagwanjee S, Rubenfeld GD. Critical care and the global burden of critical illness in adults. Lancet (London, England). 2010 Oct 16;376(9749):1339–46.

3. Iwashyna TJ. Survivorship Will Be the Defining Challenge of Critical Care in the 21st Century. Ann Intern Med. 2010 Aug 3;153(3):204.

4. The Faculty of Intensive Care Medicine. FICM Position Statement and Provisional Guidance: Recovery and Rehabilitation for Patients Following the Pandemic. 2020.

5. Johanna Josepha op't Hoog SA, Eskes AM, Johanna van Mersbergen-de Bruin MP, et al. The effects of intensive care unit-initiated transitional care interventions on elements of post-intensive care syndrome: A systematic review and meta-analysis. Aust Crit Care. 2022 May 1;35(3):309–20.

6. Hashem MD, Nallagangula A, Nalamalapu S, et al. Patient outcomes after critical illness: a systematic review of qualitative studies following hospital discharge. Crit Care. 2016;20:345.

7. Righy C, Rosa RG, Da Silva RTA, et al. Prevalence of post-traumatic stress disorder symptoms in adult critical care survivors: A systematic review and meta-analysis. Crit Care. 2019;

8. Rabiee A, Nikayin S, Hashem MD, et al. Depressive Symptoms After Critical Illness: A Systematic Review and Meta-Analysis. Crit Care Med. 2016 Sep;44(9):1744–53.

9. Nikayin S, Rabiee A, Hashem MD, et al. Anxiety symptoms in survivors of critical illness: a systematic review and meta-analysis. Gen Hosp Psychiatry. 2016 Nov;43:23–9.

10. Davydow DS, Zatzick D, Hough CL, Katon WJ. A longitudinal investigation of posttraumatic stress and depressive symptoms over the course of the year following medical-surgical intensive care unit admission. Gen Hosp Psychiatry. 2013;35(3):226–32.

11. Hatch R, Young D, Barber V, et al. Anxiety, Depression and Post Traumatic Stress Disorder after critical illness: a UK-wide prospective cohort study. Crit Care. 2018;22(1):310.

For peer review only - http://bmjopen.bmj.com/site/about/guidelines.xhtml

12. Davydow DS, Hough CL, Zatzick D, Katon WJ. Psychiatric symptoms and acute care service utilization over the course of the year following medical-surgical ICU admission: a longitudinal investigation*. Crit Care Med. 2014 Dec;42(12):2473–81.

13. McPeake J, Mikkelsen ME, Quasim T, et al. Return to Employment after Critical Illness and Its Association with Psychosocial Outcomes. A Systematic Review and Meta-Analysis. Ann Am Thorac Soc. 2019;16(10):1304–11.

14. Connolly B, Milton-Cole R, Adams C, et al. Recovery, rehabilitation and follow-up services following critical illness: an updated UK national cross-sectional survey and progress report. BMJ Open. 2021 Oct;11(10):e052214.

15. Umbrello M, Sorrenti T, Mistraletti G, et al. Music therapy reduces stress and anxiety in critically ill patients: a systematic review of randomized clinical trials. Minerva Anestesiol. 2019 Jul;85(8).

16. Jagan S, Park T, Papathanassoglou E. Effects of massage on outcomes of adult intensive care unit patients: a systematic review. Nurs Crit Care. 2019 Nov 4;24(6):414–29.

17. McIlroy PA, King RS, Garrouste-Orgeas M, et al. The Effect of ICU Diaries on Psychological Outcomes and Quality of Life of Survivors of Critical Illness and Their Relatives: A Systematic Review and Meta-Analysis. Crit Care Med. 2019;47(2):273–9.

18. Mavranezouli I, Megnin-Viggars O, Daly C, et al. Psychological treatments for post-traumatic stress disorder in adults: a network meta-analysis. Psychol Med. 2020 Mar 17;50(4):542–55.

19. Cuijpers P, Veen SC van, Sijbrandij M, et al. Eye movement desensitization and reprocessing for mental health problems: a systematic review and meta-analysis. Cogn Behav Ther. 2020 May 3;49(3):165–80.

20. Mavranezouli I, Megnin-Viggars O, Grey N, et al. Cost-effectiveness of psychological treatments for post-traumatic stress disorder in adults. PLoS One. 2020;15(4):e0232245.

21. Bisson JI, Berliner L, Cloitre M, et al. The International Society for Traumatic Stress Studies New Guidelines for the Prevention and Treatment of Posttraumatic Stress Disorder: Methodology and Development Process. J Trauma Stress. 2019 Aug 8;32(4):475–83.

22. Van Ommeren M. Guidelines for the Management of Conditions Specifically Related to Stress i WHO Library Cataloguing-in-Publication Data. 2013.

23. Ursano RJ, Carl Bell C, Eth S, et al. Practice guideline for the treatment of patients with Acute Stress Disorder and Posttraumatic Stress Disorder; Work group on ASD

For peer review only

For peer review only - http://bmjopen.bmj.com/site/about/guidelines.xhtml

and PTSD American Psychiatric Association steering committee on practice guidelines. 2010.

24. National Institute for Health and Care Excellence. Post-traumatic stress disorder: NICE guideline. NICE Guidance. 2018.

25. Haerizadeh M, Sumner JA, Birk JL, et al. Interventions for posttraumatic stress disorder symptoms induced by medical events: A systematic review. J Psychosom Res. 2020;129:109908.

26. Hulme T. Using eye movement therapy to reduce trauma after intensive care | Nursing Times. Nurs Times. 2018;114(3):18–21.

27. Clarke R. The EMDR Recent Traumatic Episode Protocol With an Intensive Care Survivor: A Case Study. J EMDR Pract Res. 2022 Apr 1;16(2):50–60.

28. Bates A, Golding H, Rushbrook S, et al. A randomised pilot feasibility study of eye movement desensitisation and reprocessing recent traumatic episode protocol, to improve psychological recovery following intensive care admission for COVID-19. J Intensive Care Soc. 2022 Nov 19;175114372211368.

29. Korn D, Maxfield L, Stickgold R, Smyth N. EMDR Fidelity Rating Scale: Version 2 | EMDR Foundation [Internet]. 2018 [cited 2021 Jun 12]. Available from: https://emdrfoundation.wpengine.com/research-grants/emdr-fidelity-rating-scale/

30. Walters SJ, Bonacho Dos Anjos Henriques-Cadby I, Bortolami O, Flight L, Hind D, Jacques RM, et al. Recruitment and retention of participants in randomised controlled trials: a review of trials funded and published by the United Kingdom Health Technology Assessment Programme. BMJ Open. 2017 Mar 20;7(3):e015276.

31. Murphy AL, Gardner DM. Pilot Testing the Theoretical Framework of Acceptability in a Process Evaluation of a Community Pharmacy–Based Men's Mental Health Promotion Program. SAGE Open. 2019 Jul 30;9(4):215824401988512.

32. Weiss DS, Marmar CR. Impact of Events Scale-Revised (IES-R).

33. Blevins CA, Weathers FW, Davis MT, et al. The Posttraumatic Stress Disorder Checklist for *DSM-5* (PCL-5): Development and Initial Psychometric Evaluation. J Trauma Stress. 2015 Dec;28(6):489–98.

34. Guy W. ECDEU assessment manual for psychopharmacology : Guy, William : Free Download, Borrow, and Streaming : Internet Archive. Rockville, Md: U.S. Dept. of Health, Education, and Welfare, Public Health Service, Alcohol, Drug Abuse, and Mental Health Administration, National Institute of Mental Health, Psychopharmacology Research Branch, Division of Extramural Research Programs; 1976.

For peer review only

For peer review only - http://bmjopen.bmj.com/site/about/guidelines.xhtml

35. Spitzer RL, Kroenke K, Williams JBW, Löwe B. A Brief Measure for Assessing Generalized Anxiety Disorder. Arch Intern Med. 2006 May 22;166(10):1092.

36. Kroenke K, Spitzer RL, Williams JB. The PHQ-9: validity of a brief depression severity measure. J Gen Intern Med. 2001 Sep;16(9):606–13.

37. Herdman M, Gudex C, Lloyd, et al. Development and preliminary testing of the new five-level version of EQ-5D (EQ-5D-5L). Qual Life Res. 2011 Dec;20(10):1727-36. doi: 10.1007/s11136-011-9903-x

38. Sim J, Lewis M. The size of a pilot study for a clinical trial should be calculated in relation to considerations of precision and efficiency. J Clin Epidemiol. 2012 Mar 1;65(3):301–8.

39. Julious SA. Sample size of 12 per group rule of thumb for a pilot study. Pharm Stat. 2005 Oct 1;4(4):287–91.

40. Skivington K, Matthews L, Simpson SA, et al. A new framework for developing and evaluating complex interventions: update of Medical Research Council guidance. BMJ. 2021 Sep 30;374:n2061.

41. Anderson R. New MRC guidance on evaluating complex interventions. BMJ. 2008 Oct 22;337:a1937.

42. Bonell C, Fletcher A, Morton M, et al. Realist randomised controlled trials: A new approach to evaluating complex public health interventions. Soc Sci Med. 2012 Dec 1;75(12):2299–306.

43. Palinkas LA, Zatzick D. Rapid Assessment Procedure Informed Clinical Ethnography (RAPICE) in Pragmatic Clinical Trials of Mental Health Services Implementation: Methods and Applied Case Study. Adm Policy Ment Health. 2019;46(2):255–70.

44. Vasileiou K, Barnett J, Thorpe S, Young T. Characterising and justifying sample size sufficiency in interview-based studies: systematic analysis of qualitative health research over a 15-year period. BMC Med Res Methodol. 2018 Dec 21;18(1):148.

45. Guetterman TC, Fetters MD, Creswell JW. Integrating Quantitative and Qualitative Results in Health Science Mixed Methods Research Through Joint Displays. Ann Fam Med. 2015 Nov 1;13(6):554–61.

46. Roberts NP, Kitchiner NJ, Kenardy J, et al. Early psychological intervention following recent trauma: A systematic review and meta-analysis. Eur J Psychotraumatol. 2019 Dec 31;10(1):1695486.

47. National Institute for Health and Care Excellence. Clinical guideline (CG83) Rehabilitation after critical illness in adults. March 2009. Accessed 14/10/2023:https://www.nice.org.uk/Guidance/CG83

For peer review only

For peer review only

For peer review only - http://bmjopen.bmj.com/site/about/guidelines.xhtml

Figure 1. **EMERALD participant timeline.** IES-R, Impact of Events Scale-Revised; QoL, Quality of Life; RCT, Randomised Controlled Trial; EMDR, Eye-Movement Desensitisation and Reprocessing; CAPS-5, Clinician Administered PTSD Scale for DSM-5

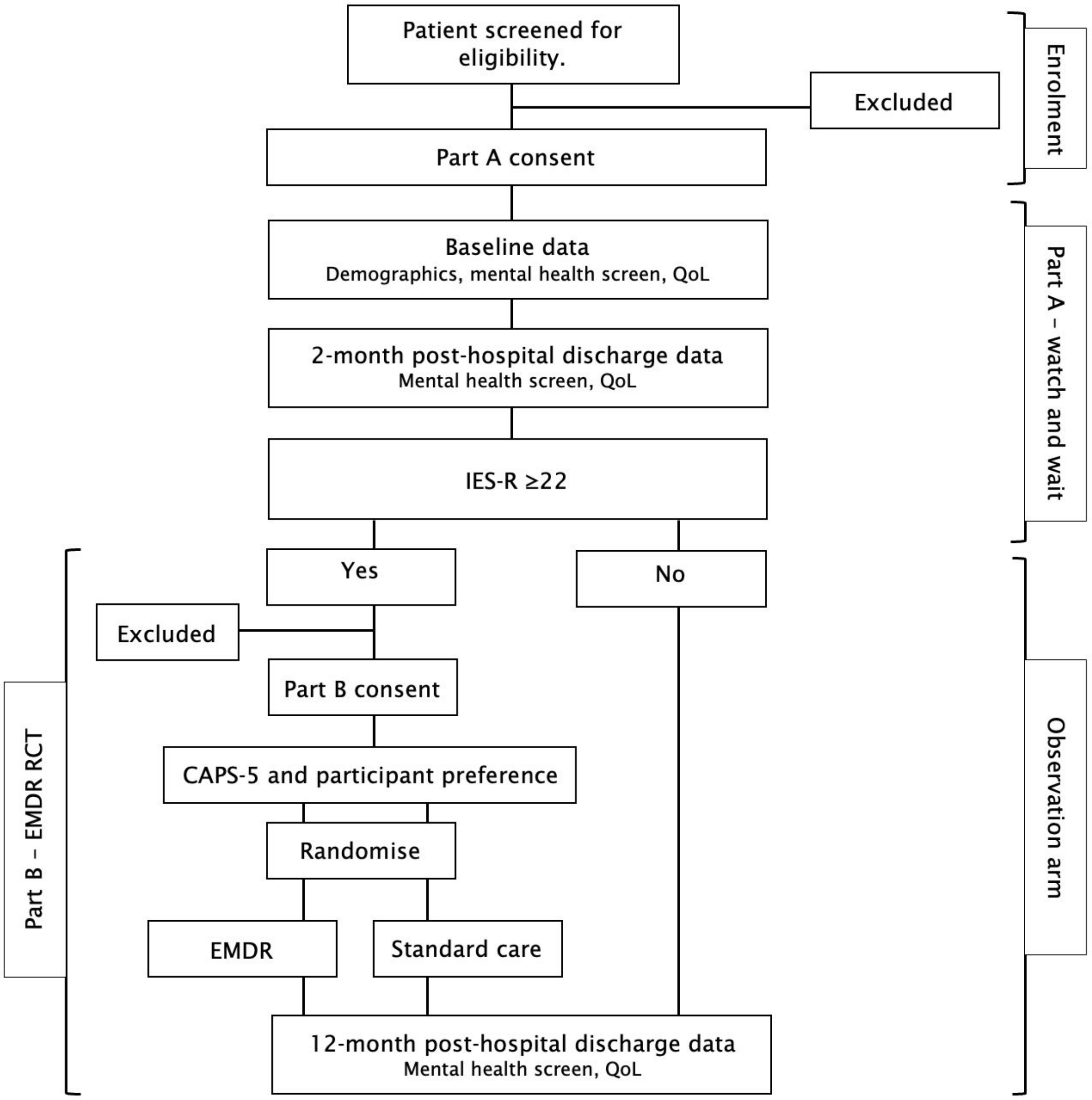

Appendix 1. EMDR treatment protocol reported according to TIDieR (Template for intervention description and replication)

Why: EMDR (Eye Movement Desensitization and Reprocessing) is hypothesised to alleviate post-traumatic symptoms among patients discharged from intensive care units (ICUs), by facilitating the adaptive processing of traumatic memories. The bilateral stimulation involved in EMDR is thought to assist in integrating memories of distressing experiences, potentially reducing the impact of trauma on mental health recovery. This study will investigate the feasibility and acceptability of delivering a randomised controlled trial (RCT) of EMDR following discharge from ICU.

What (material): No physical or informational materials were used during the intervention.

What (procedures): EMDR is a protocolised talking therapy which consists of 8 phases:

Phase 1: History taking and treatment planning: discuss participant history, with identification of traumatic events, develop a treatment plan, and assess participant's internal and external resources.

Phase 2: **Preparation:** establish a therapeutic alliance through explanation of EMDR process, discuss expectations, concerns, and questions, and equip participant with techniques to address disturbance that may arise.

Phase 3: **Assessment:** identify a target event, including associated memories, feelings, and images. Ask the participant to rate the associated disturbance, from zero to ten, using the Subjective Units of Distress scale, (SUD) and the Validity of Cognition (VOC) scale.

Phase 4: **Desensitisation:** focussing on the target event, the participant will be asked to perform side-to-side eye movements, tapping or sounds. This phase will be repeated until SUD reduces to zero or one.

Phase 5: **Installation:** Once SUD has reduced to zero-one, the participant will be guided to associate a positive belief, with the target event, until it feels consistently true.

Phase 6: **Body scan:** the participant is guided to hold both the target event and positive belief in mind, while scanning their bodily sensations from head to toe. If they identify lingering disturbance, they will repeat phase 4, until reprocessing is complete.

Phases 7 and 8 are delivered at the end of each session and are designed to ensure safety.

Phase 7: Closure: The psychological therapist assists the participant to return to a state of calm.

Phase 8: Re-evaluation: The psychological therapist and EMDR (Eye-movement desensitisation and reprocessing)

participants discuss recently processed memories and identify future target memories and directions for treatment.

Who provided: EMDR was delivered by trained, experienced psychological therapists employed by the United Kingdom (UK) National Health Service (NHS). The therapists are undergoing monthly peer to peer support and are being supervised by an EMDR Europe accredited Consultant Clinical Psychologist.

How (mode of delivery; individual or group): EMDR is delivered face-to-face or via Internet teleconference, according to participant preference.

Where: Face-to-face sessions will take place within the NHS psychological therapies clinic. Online teleconference will take place via Microsoft Teams™. Where participants are unable to attend either face-to-face or Internet sessions then a tablet with Internet dongle will be provided by the study team.

When and how much: Sessions will be delivered weekly, last for up to 60 minutes, and are provided individually. Participants will receive up to 16 sessions of EMDR.

Tailoring: The nature of trauma focused psychological therapies necessitates a personalised approach to the intervention. However representative sample of sessions will be recorded and reviewed by an expert practitioner for fidelity using the EMDR Fidelity rating scale.

How well (planned): Adherence to EMDR intervention will be expressed as a percentage of sessions offered against sessions completed. Psychological therapists will complete a diary card which will be made available to the study team at the end of the intervention.

For peer review only - http://bmjopen.bmj.com/site/about/guidelines.xhtml

Appendix 2. Secondary clinical outcome measures – description and timing

		Timepoint		
**Measure**	**Description**	**Baseline**	**Two-months post-hospital discharge**	**12-months post-hospital discharge**
**Impact of Events Scale-Revised (IES-R)** (1)	IES-R is a 22-question patient reported outcome measure (PROM) widely used to assess symptoms of PTSD in critical care research(2,3), recommended by critical care core outcome dataset developers(4-6) and the International Conference of Harmonisation of Outcome Measures(7). The 22 questions cover symptoms of intrusion, avoidance, and hyperarousal. Participants indicate how distressing the symptoms have been over the last 7 days. Symptom severity can be 0 (not at all), 1 (a little bit), 2 (moderately), 3 (quite a bit), 4 (extremely), giving a total scoring range of 0 to 88.<br>A range of cut-offs for diagnosing PTSD have been identified in different populations. To maximise sensitivity, and minimise risk of leaving PTSD untreated, we will apply the lower cut-off of 22 and retrospectively conduct a sensitivity analysis against the CAPS-5.	X	X	X
**Clinician Administered PTSD Scale for DSM-5 (CAPS-5)**(8)	CAPS-5 is a structured diagnostic interview, considered the gold-standard assessment of PTSD symptoms. In addition to evaluating the 20 symptoms listed in the DSM-5, the questions focus on the onset and duration of these symptoms, the subjective distress experienced, how these symptoms impact an individual's social and occupational functioning, any improvement in symptoms since a prior CAPS assessment, the overall validity of the responses, the severity of PTSD		X (participants who have consented to part B only)	X (participants who have consented to part B only)

For peer review only

	as a whole, and the criteria for the dissociative subtype, which encompasses depersonalization and derealization.<br>CAPS-5 assessment will be conducted face-to-face or over the phone, (according to participant preference) methods which deliver comparable results.			
**Clinical Global Impression-Severity scale (CGI-S)**(9)	The CGI-S can be used to assess symptom severity and response to treatment. It requires a clinician to rate the severity of a patient's mental illness, on a seven-point scale ranging from; 1 - normal, not at all ill, 2 - borderline mentally ill, 3 - mildly ill, 4 - moderately ill, 5 - markedly ill, 6 - severely ill, 7 - among the most extremely ill patients.		X (participants who have consented to part B only)	X (participants who have consented to part B only)
**Clinical Global Impression-Improvement scale (CGI-I)**(9)	The CGI-I requires a clinician to assess degree of improvement since baseline, in a participant's symptoms, on a seven-point ranging from; 1-very much improved; 2-much improved; 3-minimally improved; 4-no change; 5-minimally worse; 6-much worse; 7-very much worse.		X (participants who have consented to part B only)	X (participants who have consented to part B only)
**Patient Health Questionnaire (PHQ-9)**(10)	Self-administred, vaildated tool assesses depressive symptom severity(10-12). Scores are calculated by assigning 0 for 'not at all, 1- 'several days', 2 - 'more than half the days' or 3 - 'nearly every day' for responses to nine questions, giving a score in the range 0-27. PHQ-9 score of 0-4 demonstrates no - minimal depression severity. 5-9 = mild severity, 10-14 = moderate severity, 15-19 = moderately severe, 20-27 = severe.	X	X	X
**Generalised Anxiety Disorder-7 (GAD-7)**(13)	Seven-question, self-administered tool is validated to assess for anxiety symptom severity. Scores are calculated by assigning 0 for 'not at all, 1- 'several days', 2 - 'more than half the days' or 3 - 'nearly every day' for responses to nine questions, giving a score in the range 0-21. GAD-7 scores of 5, 10 and 15 represent cut-offs for mild, moderate and severe anxiety respectively.	X	X	X

For peer review only

**Health Related Quality of Life: Euroqual 5-level 5-Dimension (EQ-5D-5L)**(14)	Comprises five quality-of-life dimensions; mobility, self-care, usual activities, pain/discomfort and anxiety/depression. Participants report levels ranging from 'no problems' to 'extreme problems'.	X	X	X

For peer review only

Reporting checklist for protocol of a clinical trial.

Based on the SPIRIT guidelines.

Instructions to authors

Complete this checklist by entering the page numbers from your manuscript where readers will find each of the items listed below.

Your article may not currently address all the items on the checklist. Please modify your text to include the missing information. If you are certain that an item does not apply, please write "n/a" and provide a short explanation.

Upload your completed checklist as an extra file when you submit to a journal.

In your methods section, say that you used the SPIRITreporting guidelines, and cite them as:

Chan A-W, Tetzlaff JM, Gøtzsche PC, Altman DG, Mann H, Berlin J, Dickersin K, Hróbjartsson A, Schulz KF, Parulekar WR, Krleža-Jerić K, Laupacis A, Moher D. SPIRIT 2013 Explanation and Elaboration: Guidance for protocols of clinical trials. BMJ. 2013;346:e7586

		Reporting Item	Page Number
**Administrative information**			
Title	#1	Descriptive title identifying the study design, population, interventions, and, if applicable, trial acronym	1
Trial registration	#2a	Trial identifier and registry name. If not yet registered, name of intended registry	2
Trial registration: data set	#2b	All items from the World Health Organization Trial Registration Data Set	2 (link in text)
Protocol version	#3	Date and version identifier	n/a
Funding	#4	Sources and types of financial, material, and other support	1
Roles and responsibilities: contributorship	#5a	Names, affiliations, and roles of protocol contributors	1 and 13
Roles and	#5b	Name and contact information for the trial sponsor	n/a

For peer review only

responsibilities: sponsor contact information			
Roles and responsibilities: sponsor and funder	#5c	Role of study sponsor and funders, if any, in study design; collection, management, analysis, and interpretation of data; writing of the report; and the decision to submit the report for publication, including whether they will have ultimate authority over any of these activities	n/a
Roles and responsibilities: committees	#5d	Composition, roles, and responsibilities of the coordinating centre, steering committee, endpoint adjudication committee, data management team, and other individuals or groups overseeing the trial, if applicable (see Item 21a for data monitoring committee)	n/a
**Introduction**			
Background and rationale	#6a	Description of research question and justification for undertaking the trial, including summary of relevant studies (published and unpublished) examining benefits and harms for each intervention	2
Background and rationale: choice of comparators	#6b	Explanation for choice of comparators	2
Objectives	#7	Specific objectives or hypotheses	3
Trial design	#8	Description of trial design including type of trial (eg, parallel group, crossover, factorial, single group), allocation ratio, and framework (eg, superiority, equivalence, non-inferiority, exploratory)	3
**Methods: Participants, interventions, and outcomes**			
Study setting	#9	Description of study settings (eg, community clinic, academic hospital) and list of countries where data will be collected. Reference to where list of study sites can be obtained	4

For peer review only

Eligibility criteria	#10	Inclusion and exclusion criteria for participants. If applicable, eligibility criteria for study centres and individuals who will perform the interventions (eg, surgeons, psychotherapists)	4
Interventions: description	#11a	Interventions for each group with sufficient detail to allow replication, including how and when they will be administered	8 and supplementary material
Interventions: modifications	#11b	Criteria for discontinuing or modifying allocated interventions for a given trial participant (eg, drug dose change in response to harms, participant request, or improving / worsening disease)	8
Interventions: adherance	#11c	Strategies to improve adherence to intervention protocols, and any procedures for monitoring adherence (eg, drug tablet return; laboratory tests)	8 and supplementary material
Interventions: concomitant care	#11d	Relevant concomitant care and interventions that are permitted or prohibited during the trial	8
Outcomes	#12	Primary, secondary, and other outcomes, including the specific measurement variable (eg, systolic blood pressure), analysis metric (eg, change from baseline, final value, time to event), method of aggregation (eg, median, proportion), and time point for each outcome. Explanation of the clinical relevance of chosen efficacy and harm outcomes is strongly recommended	9
Participant timeline	#13	Time schedule of enrolment, interventions (including any run-ins and washouts), assessments, and visits for participants. A schematic diagram is highly recommended (see Figure)	see figure 1
Sample size	#14	Estimated number of participants needed to achieve study objectives and how it was determined, including clinical and statistical assumptions supporting any sample size calculations	10
Recruitment	#15	Strategies for achieving adequate participant enrolment to reach target sample size	5 and 8

Methods:

For peer review only

**Assignment of interventions (for controlled trials)**			
Allocation: sequence generation	#16a	Method of generating the allocation sequence (eg, computer-generated random numbers), and list of any factors for stratification. To reduce predictability of a random sequence, details of any planned restriction (eg, blocking) should be provided in a separate document that is unavailable to those who enrol participants or assign interventions	8
Allocation concealment mechanism	#16b	Mechanism of implementing the allocation sequence (eg, central telephone; sequentially numbered, opaque, sealed envelopes), describing any steps to conceal the sequence until interventions are assigned	8
Allocation: implementation	#16c	Who will generate the allocation sequence, who will enrol participants, and who will assign participants to interventions	8
Blinding (masking)	#17a	Who will be blinded after assignment to interventions (eg, trial participants, care providers, outcome assessors, data analysts), and how	8
Blinding (masking): emergency unblinding	#17b	If blinded, circumstances under which unblinding is permissible, and procedure for revealing a participant's allocated intervention during the trial	11
**Methods: Data collection, management, and analysis**			
Data collection plan	#18a	Plans for assessment and collection of outcome, baseline, and other trial data, including any related processes to promote data quality (eg, duplicate measurements, training of assessors) and a description of study instruments (eg, questionnaires, laboratory tests) along with their reliability and validity, if known. Reference to where data collection forms can be found, if not in the protocol	9, 10, 11
Data collection plan: retention	#18b	Plans to promote participant retention and complete follow-up, including list of any outcome data to be collected for	10,11

For peer review only

		participants who discontinue or deviate from intervention protocols	
Data management	#19	Plans for data entry, coding, security, and storage, including any related processes to promote data quality (eg, double data entry; range checks for data values). Reference to where details of data management procedures can be found, if not in the protocol	9
Statistics: outcomes	#20a	Statistical methods for analysing primary and secondary outcomes. Reference to where other details of the statistical analysis plan can be found, if not in the protocol	10
Statistics: additional analyses	#20b	Methods for any additional analyses (eg, subgroup and adjusted analyses)	na
Statistics: analysis population and missing data	#20c	Definition of analysis population relating to protocol non-adherence (eg, as randomised analysis), and any statistical methods to handle missing data (eg, multiple imputation)	na
**Methods: Monitoring**			
Data monitoring: formal committee	#21a	Composition of data monitoring committee (DMC); summary of its role and reporting structure; statement of whether it is independent from the sponsor and competing interests; and reference to where further details about its charter can be found, if not in the protocol. Alternatively, an explanation of why a DMC is not needed	12
Data monitoring: interim analysis	#21b	Description of any interim analyses and stopping guidelines, including who will have access to these interim results and make the final decision to terminate the trial	12
Harms	#22	Plans for collecting, assessing, reporting, and managing solicited and spontaneously reported adverse events and other unintended effects of trial interventions or trial conduct	11
Auditing	#23	Frequency and procedures for auditing trial conduct, if any, and whether the process will be independent from investigators and the sponsor	12
**Ethics and dissemination**			

For peer review only

Research ethics approval	#24	Plans for seeking research ethics committee / institutional review board (REC / IRB) approval	12
Protocol amendments	#25	Plans for communicating important protocol modifications (eg, changes to eligibility criteria, outcomes, analyses) to relevant parties (eg, investigators, REC / IRBs, trial participants, trial registries, journals, regulators)	12
Consent or assent	#26a	Who will obtain informed consent or assent from potential trial participants or authorised surrogates, and how (see Item 32)	5
Consent or assent: ancillary studies	#26b	Additional consent provisions for collection and use of participant data and biological specimens in ancillary studies, if applicable	na
Confidentiality	#27	How personal information about potential and enrolled participants will be collected, shared, and maintained in order to protect confidentiality before, during, and after the trial	9
Declaration of interests	#28	Financial and other competing interests for principal investigators for the overall trial and each study site	15
Data access	#29	Statement of who will have access to the final trial dataset, and disclosure of contractual agreements that limit such access for investigators	10
Ancillary and post trial care	#30	Provisions, if any, for ancillary and post-trial care, and for compensation to those who suffer harm from trial participation	11
Dissemination policy: trial results	#31a	Plans for investigators and sponsor to communicate trial results to participants, healthcare professionals, the public, and other relevant groups (eg, via publication, reporting in results databases, or other data sharing arrangements), including any publication restrictions	12
Dissemination policy: authorship	#31b	Authorship eligibility guidelines and any intended use of professional writers	12
Dissemination policy: reproducible research	#31c	Plans, if any, for granting public access to the full protocol, participant-level dataset, and statistical code	12

For peer review only

Appendices

Informed consent materials	#32	Model consent form and other related documentation given to participants and authorised surrogates	3
Biological specimens	#33	Plans for collection, laboratory evaluation, and storage of biological specimens for genetic or molecular analysis in the current trial and for future use in ancillary studies, if applicable	na

Notes:

- 2b: 2 (link in text)
- 11a: 8 and supplementary material
- 11c: 8 and supplementary material
- 13: see figure 1 The SPIRIT Explanation and Elaboration paper is distributed under the terms of the Creative Commons Attribution License CC-BY-NC. This checklist was completed on 09. November 2023 using https://www.goodreports.org/, a tool made by the EQUATOR Network in collaboration with Penelope.ai

For peer review only

BMJ Open

Protocol for a mixed methods randomised study exploring the feasibility and acceptability of Eye-Movement Desensitisation and Reprocessing for improving the mental health of traumatised survivors of intensive care following hospital discharge.

Journal:	*BMJ Open*
Manuscript ID	bmjopen-2023-081969.R1
Article Type:	Protocol
Date Submitted by the Author:	30-Nov-2023
Complete List of Authors:	Bates, Andrew; University Hospital Southampton NHS Foundation Trust, Perioperative and Critical Care Theme, NIHR Southampton Biomedical Research Centre; University of Southampton, Clinical and Experimental Sciences, Faculty of Medicine<br>Golding, Hannah; University Hospital Southampton NHS Foundation Trust, Perioperative and Critical Care Theme, NIHR Southampton Biomedical Research Centre<br>Rushbrook, Sophie; Dorset HealthCare University NHS Foundation Trust<br>Highfield, Julie; Cardiff and Vale University Health Board<br>Pattison, Natalie; University of Hertfordshire; East and North Hertfordshire NHS Trust<br>Baldwin, David ; University of Southampton Faculty of Medicine, Clinical and Experimental Sciences; Southern Health NHS Foundation Trust<br>Grocott, Michael P. W.; University Hospital Southampton NHS Foundation Trust, Perioperative and Critical Care Theme, NIHR Southampton Biomedical Research Centre; University of Southampton Faculty of Medicine, Clinical and Experimental Sciences<br>Cusack, Rebecca; Southampton University Hospitals NHS Trust, Perioperative and Critical Care Theme, NIHR Southampton Biomedical Research Centre; University of Southampton Faculty of Medicine
**Primary Subject Heading**:	Intensive care
Secondary Subject Heading:	Mental health, Rehabilitation medicine
Keywords:	PSYCHIATRY, Anxiety disorders < PSYCHIATRY, INTENSIVE & CRITICAL CARE, Feasibility Studies, MENTAL HEALTH

SCHOLARONE™
Manuscripts

Title

Protocol for a mixed methods randomised study exploring the feasibility and acceptability of Eye-Movement Desensitisation and Reprocessing for improving the mental health of traumatised survivors of intensive care following hospital discharge.

For peer review only

Authors

Andrew Bates[1,2], Hannah Golding[2], Sophie Rushbrook[3], Julie Highfield[4], Natalie Pattison[5,6], David S. Baldwin[1,7,8], Michael P W Grocott[1,2], and Rebecca Cusack[1,2]

Corresponding author: Andrew Bates; a.bates@soton.ac.uk

Abstract

Introduction

Post-traumatic symptoms are common among patients discharged from intensive care units (ICUs), adversely affecting well-being, increasing healthcare utilisation, and delaying return to work. Non-pharmacological approaches (e.g., music, therapeutic touch, patient diaries) have been suggested as candidate interventions and trauma-focused psychological interventions have been endorsed by international bodies. Neither category of intervention is supported by evidence of clinical effectiveness in patients who have been critically ill. This study assesses the feasibility and acceptability of using eye-movement desensitization and reprocessing (EMDR) to improve the mental health of ICU survivors.

Methods and analysis

EMERALD is a multicentre, two-part consent, pilot feasibility study, recruiting discharged ICU survivors from three hospitals in the United Kingdom. We are gathering demographics and measuring post-traumatic symptoms, anxiety, depression, and quality of life at baseline. Two months after discharge, participants are screened for symptoms of post-traumatic stress disorder (PTSD) using the Impact of Events Scale-Revised (IES-R). Patients with IES-R scores <22 continue in an observation arm for 12-month follow-up. IES-R scores ≥22 indicate above-threshold PTSD symptoms and trigger invitation to consent for Part B: a randomised controlled trial (RCT) of EMDR vs. usual care, with 1:1 randomisation. The study assesses feasibility (recruitment, retention, intervention fidelity) and acceptability (through semi-structured interviews), using a theoretical acceptability framework. Clinical outcomes (PTSD, anxiety, depression, and quality of life) are collected at baseline, two- and 12-months,

informing power calculations for a definitive RCT, with quantitative and qualitative data convergence guiding RCT refinements.

Ethics and dissemination

This study has undergone external expert peer review and is funded by the National Institute for Health and Care Research (Grant number: NIHR302160). Ethical approval has been granted by South Central – Hampshire A Research Ethics Committee (IRAS number: 317291) and the study is registered on ClinicalTrials.gov: NCT05591625.
Results will be disseminated through the lay media, social media, peer reviewed publication and conference presentation.

Keywords

Critical care; Eye Movement Desensitization and Reprocessing; stress disorder, post-traumatic; anxiety

Article summary

Strengths and limitations of this study

- Adheres to Medical Research Council guidance for evaluating complex healthcare interventions.
- Mixed methods probe feasibility and acceptability enabling us to address cultural and contextual factors.
- Consistent with existing clinical pathways and best practice guidance.
- Not powered to detect between-group, clinically significant differences in post-traumatic symptoms.

Introduction

Background and rationale

Critically ill patients in intensive care units (ICUs) receive life-saving treatment, yet the burden of long-term physical, cognitive, and mental health issues, collectively known as 'post-intensive care syndrome', is significant(1). Global ICU admissions are on the rise(2) and there is growing recognition of the need to address post-ICU survivorship as a defining challenge in 21st-century intensive care medicine(3). Despite this, healthcare providers often overlook this phase(4), resulting in multiple care transitions away from clinicians with an understanding of the underlying aetiology(5).

Amidst the existential threat of critical illness, patients endure invasive treatments, potent psychoactive drugs, a busy and confusing environment, and limited communication, leading to normal acute anxiety responses(6). However, a substantial proportion continue to suffer unpleasant psychological and somatic symptoms. Post-ICU discharge, 20-25% experience symptoms similar to those of post-traumatic stress disorder (PTSD) (7), with over 30% and 40% experiencing depression(8) and anxiety(9), respectively. These symptoms can be

For peer review only

persistent(10), co-occurring(11), and are associated with adverse outcomes including reduced quality of life, increased healthcare utilisation and delayed return to work(9,12,13).

Despite this, access to clinical psychology remains underrepresented in United Kingdom (UK) ICU recovery services(14). Interventions like music therapy(15), therapeutic touch(16), and patient diaries(17) have been explored, but systematic reviews reveal that definitive evidence of long-term effect is lacking. Trauma-focused psychological therapies, such as Eye Movement Desensitization and Reprocessing (EMDR), offer some promise, with meta-analyses showing significant reductions in PTSD, anxiety, and depression for treating a diverse range of traumatised populations(18,19). EMDR is cost-effective(20) and is internationally recommended by major organisations for trauma-related symptoms(21–24).

Recent investigations of EMDR's effectiveness in treating medical event-induced trauma, following cancer, stroke, cardiac events, and multiple sclerosis have yielded promising but inconclusive findings(25). Case studies with ICU survivors(26,27) and our own novel work with survivors of coronavirus disease (COVID-19) related critical illness(28) also show promise, underscoring the need for systematic evaluation in this population. However, definitive evidence of benefit is not available.

Objectives:

The primary objective of the EMERALD study is to evaluate the feasibility and acceptability of an EMDR intervention for adult patients displaying traumatic stress symptoms following ICU discharge. These findings will guide the design of a robust, fully powered randomised controlled trial (RCT), aligning with Medical Research Council (MRC) guidance on evaluating complex medical interventions. Secondary clinical outcomes will inform the selection of a primary outcome for the larger trial and provide variance estimates for sample size calculations. Additionally, a light-touch observation arm will offer insights into the mental health trajectory of ICU survivors without traumatic stress symptoms two-months after hospital discharge.

Methods: Participants, interventions, and outcomes

Design

This is a multicentre, mixed methods, randomised controlled pilot feasibility study, with a two-part consent process and is reported using the Standard Protocol Items: Recommendations for Interventional Trials (SPIRIT) reporting guidelines(29)(supplemental file 1: Reporting checklist for protocol of a clinical trial). Initially, all participants enter Part A, which is an observational study, where they complete a series of mental health questionnaires at baseline, two-months, and 12-months post-hospital discharge. If a participant shows symptoms of post-traumatic stress at the two-month mark (scoring ≥22 on the Impact Events Scale-Revised (IES-R)), they are invited to consider participating in Part B, which is an interventional study of EMDR vs. standard care. Those without post-traumatic

For peer review only - http://bmjopen.bmj.com/site/about/guidelines.xhtml

stress symptoms at 2 months (≤21 on the IES-R) or those who decline participation in Part B will be offered continuation of the observation arm. All participants from both Part A and Part B repeat the study assessments at 12 months post-hospital discharge. See Figure 1 for the participant timeline.

Figure 1. **EMERALD participant timeline.** IES-R, Impact of Events Scale-Revised; QoL, Quality of Life; RCT, Randomised Controlled Trial; EMDR, Eye-Movement Desensitisation and Reprocessing; CAPS-5, Clinician Administered PTSD Scale for DSM-5

Study setting

The study is sponsored by the University Hospital Southampton National Health Service (NHS) Foundation Trust (FT). Recruitment will occur after adult patients are discharged from three adult NHS ICUs in the UK: University Hospital Southampton, Royal Bournemouth Hospital, and Poole General Hospital. The intervention will be provided through NHS psychological therapy services in proximity to the study participants, specifically Southern Health NHS FT and Dorset Healthcare University NHS FT.

Part A participant recruitment

Recruitment is anticipated to occur between February 2023 and May 2024. Eligibility screening will target consecutive patients discharged from the participating ICUs. Research staff will approach eligible patients on hospital wards or within two months following hospital discharge, via a telephone call or email, providing a participant information sheet. Patients will be invited to complete an informed consent form (ICF), accessible electronically through Qualtrics™ on tablet devices provided by the trial team, via an emailed link or on paper to suit patient preference. This initial consent pertains to their participation in the observational study (Part A), involving baseline data collection and psychometric assessments, with a follow-up evaluation at two months and 12-months following hospital discharge.

Eligibility criteria

Eligibility will be determined by hospital research nurses acting under delegated authority of the local Principal Investigator. Patients will be eligible for part A if they meet the following criteria:

- Survivor of an intensive care admission, who received level 3 care for >24 hours.
- Aged ≥18 years.
- Capacity to provide informed consent.

For peer review only - http://bmjopen.bmj.com/site/about/guidelines.xhtml

	Baseline	2 months post-discharge	3-9 months post-discharge	12-months post-discharge
**Informed Consent**	X<br>Part A	X*<br>Part B		
**Demographics**	X			
**IES-R**	X	X		X

Patients will be excluded if they meet any of the following criteria:

- Pre-existing cognitive impairment such as dementia.
- Pre-existing diagnosis of psychosis.
- Not expected to survive beyond hospital discharge.
- Traumatic brain injury.

Baseline data collection

Research staff will collect demographic data, medical history, and ICU admission history following consent. All participants will complete the Impact of Events Scale-Revised (IES-R), Patient Health Questionnaire-9, (PHQ-9) Generalised Anxiety Disorder 7, (GAD-7) and the Euroqol 5 Dimension 5 Level. (EQ-5D-5L)

Two-month post-hospital discharge assessment

All participants will be requested to repeat the IES-R, PHQ-9, GAD-7, and EQ-5D-5L. These patient-reported outcome measures can be completed electronically via an emailed link or by using paper versions sent with a prepaid return envelope.

The study team will review the IES-R responses. Participants with a total score ≥22, indicative of post-traumatic stress symptoms, will be approached to consider participation in an EMDR vs. usual care RCT (Part B).

Participants without symptoms (IES-R≤21) or those not interested or unable to participate in the RCT will continue in the observational study, completing the 12-month follow-up assessment.

12-month follow-up assessment

Research staff will ask all participants, in both the observation group (Part A only) and RCT (Part A and Part B), to repeat the IES-R, PHQ-9, GAD-7 and the EQ-5D-5L, at 12-months post-hospital discharge. See Table 1 for the full study schedule of events.

For peer review only - http://bmjopen.bmj.com/site/about/guidelines.xhtml

**CAPS-5, CGI-S***		X*		X*
**PHQ-9**	X	X		X
**GAD-7**	X	X		X
**EQ-5D-5L**	X	X		X
**EMDR Intervention**			X*	
**IES-R, PHQ-9, GAD-7 (EMDR group only)**				
**Randomisation preference***		X*		
**Process Evaluation**			X	X

Table 1: EMERALD study schedule of events. X* for participants consenting to part B of the study only.

For peer review only

Part B participant recruitment

Participants scoring ≥22 on the 2-month IES-R will receive a phone call or email from the study team, inviting them to consider consenting to Part B, the EMDR vs. usual care RCT. The Part B PIS and ICF will be accessible electronically or via postal delivery. Those who consent to Part B will first undergo a Clinician Administered PTSD Scale for Diagnostic and Statistical Manual of Mental Disorders, fifth edition (CAPS-5) assessment to evaluate PTSD symptoms and a Clinical Global Impression of Illness Severity (CGI-S) assessment with the Chief Investigator (CI). Additionally, participants will be asked to rate their preference for study arm strength using a Likert scale ranging from 0 to 10.

Randomisation

Consenting participants will be randomly assigned to either receive usual care or usual care combined with EMDR, utilising an internet-based system, following their CAPS-5 assessment. A researcher outside of the study team will undertake randomisation, to ensure the CI remains blinded to study group allocation. Random allocation will occur in a 1:1 ratio, designating them to the control group (CG) for usual care or the intervention group (EMDR) for usual care plus EMDR.

Control Group (CG): Participants in the control group will receive the standard care package prescribed upon hospital discharge, which may vary across study hospitals. Variations in standard care will be investigated through qualitative process evaluation and reported in the results manuscript. In case of adverse physical or psychological health conditions, they will access care through the usual available channels.

Intervention Group (EMDR): Participants randomised to the intervention group will receive the standard clinical care package following hospital discharge. Additionally, they will be referred to a participating adult NHS Psychological Therapies service using the established NHS-NHS referral system, identifying them as EMERALD participants. NHS Psychology teams will adhere to this research protocol for treatment. Any deviations from the protocol will be reported to the study team.

EMDR sessions, whether conducted via videoconference or face-to-face, will ideally commence within 4 weeks of referral and will be administered by trained EMDR therapists, who are supervised by a Consultant Clinical Psychologist. EMDR comprises eight phases, providing a structured treatment framework that supports consistency in session effects. The protocolised nature of EMDR facilitates training and replication in controlled studies. With participant and therapist agreement, some sessions will be recorded and assessed using the EMDR Fidelity Rating Scale (EFRS)(30) to allow granular reporting of the delivered intervention. The EMDR protocol, reported according to the TIDieR (Template for intervention description and replication) guidelines, is available in supplemental file 2. Sessions will last up to 60 minutes, and therapist-recorded adherence will track the number of sessions offered versus those completed. Participants may receive up to 16 EMDR sessions, based on the therapist's ongoing assessment of need.

Outcome measures

Primary outcome measures are feasibility and acceptability of trial process, to participants and staff.

Feasibility will be reported using the CONSORT statement as:

- Recruitment rate part A – we anticipate an average recruitment of 10 patients per month across the three participating sites. This is well above the median recruitment of 0.95 participants recruited per site per month, reported in a review of trials listed in the NIHR journals library (1997-2020)(31).

For peer review only - http://bmjopen.bmj.com/site/about/guidelines.xhtml

- Consent rate – number of patients recruited, expressed as a percentage of patients approached. Based on our previous work we expect this to be greater than 30%(28).
- Adherence will be determined by completion of ≥75% of planned EMDR sessions completed.
- Retention will be determined by ≥75% of participants completing the study follow-up assessment.

Acceptability will be determined by a qualitative process evaluation using semi-structured interviews, and reported according to the Theoretical Framework of Acceptability(32). In addition, we will assess fidelity to the EMDR delivery model using the EMDR fidelity rating scale (EFRS). This will enable us to account for variability in intervention delivery. Safety will be determined by assignment of causality of serious events. Events attributable to trial procedures will be reviewed by trial management board, study sponsor and the research ethics committee, to determine ongoing feasibility.

In addition to sociodemographic characteristics, and medical history (including ICU admission data) secondary outcome measures will be collected at baseline, 2-months, and 12-months post-hospital discharge to capture possible clinical outcomes, mediators, moderators, and covariates that may be included in the subsequent, definitive effectiveness trial. A detailed description of each of these measures is provided in supplemental file 3. All data will be stored securely, pseudonymised by study number, on the Qualtrics^{TM} electronic database. The secondary outcome measures include:

- Change in PTSD symptom severity using the Impact of Events Scale – Revised (IES-R)(33)
- Change in categorical diagnosis of PTSD using IES-R.
- Post-traumatic stress score using Clinician administered PTSD scale for DSM-5 (CAPS-5)(34)
- Clinical Global Impression-Severity scale (CGI-S)(35)
- Sensitivity analysis: to determine whether PTSD symptom burden identified by IES-R corresponds with those identified by CAPS-5.
- Anxiety: Generalised Anxiety Disorder-7 (GAD-7)(36)
- Depression: Patient Health Questionnaire-9 (PHQ-9)(37)
- Quality of life EuroQol Five Dimension- Five level scale (EQ5D -5L)(38)
- Clinical Global Impression of Improvement (CGI-I)(35)

Sample size

As this is a feasibility study, an *a priori* sample size calculation is not applicable. The findings will guide the sample size determination for a potential definitive RCT. Sample sizes of feasibility studies between 24 and 50 have been recommended, to provide adequate estimate of standard deviation for sample size calculation(39,40).

For peer review only - http://bmjopen.bmj.com/site/about/guidelines.xhtml

To achieve this, a total of 160 patients will be enrolled in Part A to assess feasibility adequately. Based on an expected incidence of 20-25% post-ICU PTSD, we anticipate that around 40 patients will proceed to the Part B RCT with an IES-R PTSD score ≥22. The remaining 120 participants will continue in the observation arm, with a 12-month re-assessment. Accounting for an estimated 25% mortality or loss to follow-up across all study arms, we anticipate approximately 30 participants completing the RCT and 90 participants completing the observation arm.

Data plan and analysis

Recruitment, retention, and trial completion data will be visually represented in a CONSORT diagram. Quantitative outcome analysis, encompassing measures such as IES-R, CAPS-5, PHQ-9, GAD-7, and EQ5D-5L, will primarily be descriptive, emphasizing estimation. Baseline measures and outcomes will be summarised using appropriate descriptive statistics, complete with associated confidence intervals. The focus of interpretation will centre on the implications of these results for the feasibility of the main trial. Furthermore, we will conduct a confirmatory factor analysis (CFA) of the DSM-5's four-factor PTSD diagnostic criteria, utilising data pooled from the CAPS-5 interviews.

Qualitative process evaluation

Qualitative description will be employed to construct a comprehensive overview of participants' and staff perceived experiences and the impact of the EMERALD study. This includes assessing the perceived burden associated with study participation and undertaking research activities. Qualitative interview data will serve to validate, elaborate upon, and broaden our understanding of the study's acceptability and feasibility, while also shedding light on potential factors that may hinder or enhance the EMERALD study. This information will be invaluable in refining the design of the subsequent RCT.

Method for obtaining and evaluating qualitative data.

The process evaluation aligns with MRC guidance for complex intervention evaluations(41). However, this guidance has faced criticism for its lack of theory-driven approaches(42), potentially leading to limited insight into contextual factors and mechanisms of change(43). To efficiently capture implementation processes, we will employ Rapid Assessment Procedure Informed Clinical Ethnography(44).

Stage 1- data collection involves selecting a purposive, diverse sample of trial participants and psychological therapists, minimizing bias by adapting the sample to study needs. Participants will be invited for recorded telephone or videoconference interviews at their convenience. We will use semi-structured interviews guided by relevant objectives, incorporating patient and public involvement (PPI) recommendations, recent literature, and a systematic review. See supplementary material 4 for participant interview guide and

For peer review only - http://bmjopen.bmj.com/site/about/guidelines.xhtml
For peer review only

psychological therapist interview guide. Sampling will continue until data saturation is reached, typically with 15-20 interviews(45). The questions will be open-ended, and we will take field notes while digitally recording and transcribing interviews. The data will be reviewed by a senior researcher within the team, to assess the need for further data collection.

Stage 2 - the anonymised dataset will be securely stored and analysed using NVivo™ qualitative data software. The analysis will follow the theoretical framework of acceptability, deductively coding content into seven constructs(32); affective attitude, burden, intervention coherence, ethicality, opportunity costs, perceived effectiveness, and self-efficacy.

Preliminary interpretation of emerging themes will be independently conducted, with consensus reached through discussion. Additional data collection will be considered if necessary. Agreed findings will be presented to a sample of study participants and PPI representatives to ensure validity and comprehensiveness.

Stage 3 - will integrate qualitative findings with quantitative RCT data during the post-study interpretation phase. We will map data using a mixed methods joint display(46), and providing a holistic understanding of predetermined study objectives following established principles.

Safety considerations

Several systematic reviews have reported no adverse events attributable to EMDR. The intervention will be undertaken by suitably trained and experienced psychological therapists employed by the NHS. The service has an established and defined risk management and clinical governance structure. Online sessions will be compliant with Digital Approaches to therapy guidance from the British Psychological Society and NHS Digital. (This guidance contains expected standards relating to safeguarding, information governance, and GDPR).

Participants who exhibit symptoms of intrusion/ escalation will be treated according to the protocol unless it is determined that further treatment or escalation to emergency care may be necessary/ indicated. If further treatment is required, the most appropriate course of action and referral pathway will be decided on a case-by-case basis by the psychology team. If deemed necessary the Chief Investigator will be unblinded to group allocation, to contribute to the safety discussion.

For peer review only - http://bmjopen.bmj.com/site/about/guidelines.xhtml

Monitoring and trial oversight

Day to day management will be the joint responsibility of the Chief Investigator, Senior Project Co- Ordinator and Co-Investigators. This project is part of a PhD study undertaken by Andrew Bates (CI) with supervision by the co-investigators and authors.

Monitoring: The CI will facilitate monitoring by the local quality manager, Research Ethics Committee (REC) review and provide access to source data as required. Following any monitoring a report will be provided which will summarise the visit and documents, along with any findings. The CI will be responsible for ensuring that all findings are addressed appropriately. The study group will review all events in a timely manner. Additional monitoring will be scheduled where there is evidence of suspicion of non-compliance with the study protocol.

Patient and public involvement

Patient and public involvement (PPI) has shaped the study design, and this collaboration will persist throughout the project in the following ways:

Patient Advisory Group: An established PPI group attended advisory group meetings during project development. We are planning for meetings to occur every six months to review research findings, discuss key points, review press releases and dissemination outputs. Any study design amendments will be discussed and approved before submission.

Study Management Steering Group: Two PPI members will serve as patient representatives in this decision-making group. They will oversee trial progress, review findings and outputs, approve project changes, and address arising issues, conflicts, and risks in three meetings per year. One PPI group member will attend an Intensive Care conference to co-present study findings to clinical and academic leaders.

Patient Groups and Third Sector: Study findings and dissemination outputs will be shared with and reviewed by patient groups and organisations such as ICU Steps, EMDR UK, EMDR Europe, and Anxiety UK. This ensures the inclusion of the patient perspective in the manuscript and keeps relevant stakeholders well-informed.

Meetings will be conducted face-to-face with the option of videoconferencing for accessibility. A plain English research report, agenda, and previous minutes will be circulated before each meeting, and meetings may be recorded with participant consent for later reference. Ongoing training tailored to individual needs will be provided for all participants, and the Public Involvement Lead for South Central Research Design Service will oversee ongoing PPI efforts.

For peer review only - http://bmjopen.bmj.com/site/about/guidelines.xhtml

Ethics and dissemination

This study obtained prior approval from the South Central - Hampshire A Research Ethics Committee (REC) (22/SC/0410) before approaching participants, who will also review protocol modifications. Ethics approval covers all NHS trial sites, which were activated before enrolling patients.

The trial will adhere to the principles outlined in the 18th World Medical Assembly's recommendations from Helsinki 1964, as revised and recognized by governing laws and EU Directives. Consent to participate in the trial will be obtained only after providing a comprehensive explanation of treatment options, including conventional and widely accepted methods. The right of individuals to decline participation without specifying reasons will be respected.

Once a participant is enrolled in the trial, clinicians may administer alternative treatments beyond the protocol if they deem it in the participant's best interest, with the reasons duly documented. The participant will continue within the trial for follow-up and data analysis based on their allocated treatment option. Likewise, participants are free to withdraw from protocol treatment and trial follow-up at any time without providing reasons, without affecting their subsequent treatment.

The Chief Investigator will inform the REC upon study completion. In cases of premature termination, the CI will promptly notify the REC, including the reasons for the early conclusion.

Within one year following the study's conclusion, the CI will submit a final report containing results and any related publications or abstracts to the REC.

Dissemination activities will include but not be limited to:

- Publication in peer reviewed journals.
- Feedback to PPI study focus group.
- Feedback to study participants.
- Presentations to local clinical teams and managers and commissioners.
- Presentation at international conferences and within inter-disciplinary clinical networks.
- Public webinars, digital and social media.

Discussion

The EMERALD study represents the second phase of our innovative exploration into whether EMDR can alleviate psychological distress after ICU discharge. Our mixed methods approach, in line with MRC guidance for assessing complex healthcare interventions, enhances the study's robustness(41). It allows us to capture cultural and contextual factors often missed in purely quantitative designs, thus improving the reliability of our findings, and informing the design of our upcoming definitive RCT.

For peer review only - http://bmjopen.bmj.com/site/about/guidelines.xhtml

Building on the lessons from our prior study, CovEMERALD(28), we have incorporated screening for psychological distress before entry into the RCT, aligning with recent review recommendations(47). Adopting a two-months post-hospital discharge screening for PTSD follows both ICU rehabilitation(48) and PTSD treatment guidelines(24). Furthermore, participants have the flexibility to choose either face-to-face or online intervention, without challenging participants' physical or psychological vulnerabilities.

A noteworthy aspect of this project is the strong collaboration between clinical academics specialising in intensive care, psychiatry, and psychology, bolstered by our patient representatives, individuals with valuable lived experiences.

It is important to interpret clinical findings from this study cautiously, as it is not powered to detect clinically significant differences between groups. Nevertheless, these outcomes will inform future power calculations for the definitive RCT.

For peer review only

Author affiliations

[1]Clinical and Experimental Sciences, Faculty of Medicine, University of Southampton, UK

[2] Perioperative and Critical Care Theme, NIHR Southampton Biomedical Research Centre, University Hospital Southampton / University of Southampton, Southampton, UK

[3]Dorset Healthcare University National Health Service Foundation Trust

[4]Cardiff and Vale University Health Board, UK

[5]University of Hertfordshire

[6]East and North Hertfordshire NHS Trust

[7]Southern Health NHS Foundation Trust

[8]University Department of Psychiatry and Mental Health, University of Cape Town, South Africa

Twitter

Andrew Bates @AndyCBates and Michael Grocott @mike_grocott

ORCID iDs

Andrew Bates https://orcid.org/0000-0002-3614-0270
Michael Grocott https://orcid.org/0000-0002-9484-7581

Acknowledgements

We acknowledge the contribution of our public and patient involvement (PPI) team. Thank you to the NHS intervention delivery teams for helping to design and deliver the EMDR; Janet Hathaway, Tara Walker, Shalini Ramani, Vanessa Ballard, Emer Young, Michelle Gormley, and Rachel Sadler. Thanks to the research nurses at recruiting sites; Jonathan Biss, Karen Salmon, Sally Pitts, Debbie Branney, Yasmin De'Ath, and Emma Langridge. Thanks also to the nurses leading the ICU follow-up services who understand and appreciate the complex convalescence faced by our patients; Fiona Hall, Rachael Hopkins, and Gemma Turnbull. AB would like to thank Dr Kay Mitchell, Professor Robert Crouch, the team at

For peer review only - http://bmjopen.bmj.com/site/about/guidelines.xhtml

Southampton Academy of Research, Research Support Service South Central and University Hospital Southampton Research and Development for their support with the NIHR Clinical Doctoral Research Fellowship application and ongoing studies.

Funding

National Institute for Health and Care Research (Grant number: NIHR302160).

Competing interests

None declared.

Provenance and peer review

This work has undergone expert external peer review by the NIHR Clinical Doctoral Research Fellowship panel. It has undergone expert internal peer review by study sponsors at University Hospital Southampton.

Authors contributions

AB and SR conceived the research idea. AB developed the theory, research plan, drafted the manuscript and is Chief Investigator for the study. HG acted as project manager during study set-up. All authors (AB, HG, SR, JH, NP, DSB, MG, and RC) contributed to the study development and have reviewed, revised, and approved this manuscript.

Word count: 3443

References

1. Desai S V., Law TJ, Needham DM. Long-term complications of critical care. Crit Care Med. 2011 Feb;39(2):371–9.

2. Adhikari NKJ, Fowler RA, Bhagwanjee S, Rubenfeld GD. Critical care and the global burden of critical illness in adults. Lancet (London, England). 2010 Oct 16;376(9749):1339–46.

3. Iwashyna TJ. Survivorship Will Be the Defining Challenge of Critical Care in the 21st Century. Ann Intern Med. 2010 Aug 3;153(3):204.

4. The Faculty of Intensive Care Medicine. FICM Position Statement and Provisional Guidance: Recovery and Rehabilitation for Patients Following the Pandemic. 2020.

5. Johanna Josepha op't Hoog SA, Eskes AM, Johanna van Mersbergen-de Bruin MP, et

For peer review only - http://bmjopen.bmj.com/site/about/guidelines.xhtml

al. The effects of intensive care unit-initiated transitional care interventions on elements of post-intensive care syndrome: A systematic review and meta-analysis. Aust Crit Care. 2022 May 1;35(3):309–20.

6. Hashem MD, Nallagangula A, Nalamalapu S, et al. Patient outcomes after critical illness: a systematic review of qualitative studies following hospital discharge. Crit Care. 2016;20:345.

7. Righy C, Rosa RG, Da Silva RTA, et al. Prevalence of post-traumatic stress disorder symptoms in adult critical care survivors: A systematic review and meta-analysis. Crit Care. 2019;

8. Rabiee A, Nikayin S, Hashem MD, et al. Depressive Symptoms After Critical Illness: A Systematic Review and Meta-Analysis. Crit Care Med. 2016 Sep;44(9):1744–53.

9. Nikayin S, Rabiee A, Hashem MD, et al. Anxiety symptoms in survivors of critical illness: a systematic review and meta-analysis. Gen Hosp Psychiatry. 2016 Nov;43:23–9.

10. Davydow DS, Zatzick D, Hough CL, Katon WJ. A longitudinal investigation of posttraumatic stress and depressive symptoms over the course of the year following medical-surgical intensive care unit admission. Gen Hosp Psychiatry. 2013;35(3):226–32.

11. Hatch R, Young D, Barber V, et al. Anxiety, Depression and Post Traumatic Stress Disorder after critical illness: a UK-wide prospective cohort study. Crit Care. 2018;22(1):310.

12. Davydow DS, Hough CL, Zatzick D, Katon WJ. Psychiatric symptoms and acute care service utilization over the course of the year following medical-surgical ICU admission: a longitudinal investigation*. Crit Care Med. 2014 Dec;42(12):2473–81.

13. McPeake J, Mikkelsen ME, Quasim T, et al. Return to Employment after Critical Illness and Its Association with Psychosocial Outcomes. A Systematic Review and Meta-Analysis. Ann Am Thorac Soc. 2019;16(10):1304–11.

14. Connolly B, Milton-Cole R, Adams C, et al. Recovery, rehabilitation and follow-up services following critical illness: an updated UK national cross-sectional survey and progress report. BMJ Open. 2021 Oct;11(10):e052214.

15. Umbrello M, Sorrenti T, Mistraletti G, et al. Music therapy reduces stress and anxiety in critically ill patients: a systematic review of randomized clinical trials. Minerva Anestesiol. 2019 Jul;85(8).

16. Jagan S, Park T, Papathanassoglou E. Effects of massage on outcomes of adult intensive care unit patients: a systematic review. Nurs Crit Care. 2019 Nov

For peer review only

For peer review only - http://bmjopen.bmj.com/site/about/guidelines.xhtml

4;24(6):414–29.

17. McIlroy PA, King RS, Garrouste-Orgeas M, et al. The Effect of ICU Diaries on Psychological Outcomes and Quality of Life of Survivors of Critical Illness and Their Relatives: A Systematic Review and Meta-Analysis. Crit Care Med. 2019;47(2):273–9.

18. Mavranezouli I, Megnin-Viggars O, Daly C, et al. Psychological treatments for post-traumatic stress disorder in adults: a network meta-analysis. Psychol Med. 2020 Mar 17;50(4):542–55.

19. Cuijpers P, Veen SC van, Sijbrandij M, et al. Eye movement desensitization and reprocessing for mental health problems: a systematic review and meta-analysis. Cogn Behav Ther. 2020 May 3;49(3):165–80.

20. Mavranezouli I, Megnin-Viggars O, Grey N, et al. Cost-effectiveness of psychological treatments for post-traumatic stress disorder in adults. PLoS One. 2020;15(4):e0232245.

21. Bisson JI, Berliner L, Cloitre M, et al. The International Society for Traumatic Stress Studies New Guidelines for the Prevention and Treatment of Posttraumatic Stress Disorder: Methodology and Development Process. J Trauma Stress. 2019 Aug 8;32(4):475–83.

22. Van Ommeren M. Guidelines for the Management of Conditions Specifically Related to Stress i WHO Library Cataloguing-in-Publication Data. 2013.

23. Ursano RJ, Carl Bell C, Eth S, et al. Practice guideline for the treatment of patients with Acute Stress Disorder and Posttraumatic Stress Disorder; Work group on ASD and PTSD American Psychiatric Association steering committee on practice guidelines. 2010.

24. National Institute for Health and Care Excellence. Post-traumatic stress disorder: NICE guideline. NICE Guidance. 2018.

25. Haerizadeh M, Sumner JA, Birk JL, et al. Interventions for posttraumatic stress disorder symptoms induced by medical events: A systematic review. J Psychosom Res. 2020;129:109908.

26. Hulme T. Using eye movement therapy to reduce trauma after intensive care | Nursing Times. Nurs Times. 2018;114(3):18–21.

27. Clarke R. The EMDR Recent Traumatic Episode Protocol With an Intensive Care Survivor: A Case Study. J EMDR Pract Res. 2022 Apr 1;16(2):50–60.

28. Bates A, Golding H, Rushbrook S, et al. A randomised pilot feasibility study of eye movement desensitisation and reprocessing recent traumatic episode protocol, to improve psychological recovery following intensive care admission for COVID-19. J

For peer review only

Intensive Care Soc. 2022 Nov 19;175114372211368.

29. Chan A-W, Tetzlaff JM, Gøtzsche PC, et al. SPIRIT 2013 explanation and elaboration: guidance for protocols of clinical trials. BMJ. 2013 Jan 8;346:e7586.

30. Korn D, Maxfield L, Stickgold R, Smyth N. EMDR Fidelity Rating Scale: Version 2 | EMDR Foundation [Internet]. 2018 [cited 2021 Jun 12]. Available from: https://emdrfoundation.wpengine.com/research-grants/emdr-fidelity-rating-scale/

31. Walters SJ, Bonacho Dos Anjos Henriques-Cadby I, Bortolami O, Flight L, Hind D, Jacques RM, et al. Recruitment and retention of participants in randomised controlled trials: a review of trials funded and published by the United Kingdom Health Technology Assessment Programme. BMJ Open. 2017 Mar 20;7(3):e015276.

32. Murphy AL, Gardner DM. Pilot Testing the Theoretical Framework of Acceptability in a Process Evaluation of a Community Pharmacy–Based Men's Mental Health Promotion Program. SAGE Open. 2019 Jul 30;9(4):215824401988512.

33. Weiss DS, Marmar CR. Impact of Events Scale-Revised (IES-R).

34. Blevins CA, Weathers FW, Davis MT, et al. The Posttraumatic Stress Disorder Checklist for *DSM-5* (PCL-5): Development and Initial Psychometric Evaluation. J Trauma Stress. 2015 Dec;28(6):489–98.

35. Guy W. ECDEU assessment manual for psychopharmacology : Guy, William : Free Download, Borrow, and Streaming : Internet Archive. Rockville, Md: U.S. Dept. of Health, Education, and Welfare, Public Health Service, Alcohol, Drug Abuse, and Mental Health Administration, National Institute of Mental Health, Psychopharmacology Research Branch, Division of Extramural Research Programs; 1976.

36. Spitzer RL, Kroenke K, Williams JBW, Löwe B. A Brief Measure for Assessing Generalized Anxiety Disorder. Arch Intern Med. 2006 May 22;166(10):1092.

37. Kroenke K, Spitzer RL, Williams JB. The PHQ-9: validity of a brief depression severity measure. J Gen Intern Med. 2001 Sep;16(9):606–13.

38. Herdman M, Gudex C, Lloyd, et al. Development and preliminary testing of the new five-level version of EQ-5D (EQ-5D-5L). Qual Life Res. 2011 Dec;20(10):1727-36. doi: 10.1007/s11136-011-9903-x

39. Sim J, Lewis M. The size of a pilot study for a clinical trial should be calculated in relation to considerations of precision and efficiency. J Clin Epidemiol. 2012 Mar 1;65(3):301–8.

40. Julious SA. Sample size of 12 per group rule of thumb for a pilot study. Pharm Stat. 2005 Oct 1;4(4):287–91.

For peer review only - http://bmjopen.bmj.com/site/about/guidelines.xhtml

For peer review only

41. Skivington K, Matthews L, Simpson SA, et al. A new framework for developing and evaluating complex interventions: update of Medical Research Council guidance. BMJ. 2021 Sep 30;374:n2061.

42. Anderson R. New MRC guidance on evaluating complex interventions. BMJ. 2008 Oct 22;337:a1937.

43. Bonell C, Fletcher A, Morton M, et al. Realist randomised controlled trials: A new approach to evaluating complex public health interventions. Soc Sci Med. 2012 Dec 1;75(12):2299–306.

44. Palinkas LA, Zatzick D. Rapid Assessment Procedure Informed Clinical Ethnography (RAPICE) in Pragmatic Clinical Trials of Mental Health Services Implementation: Methods and Applied Case Study. Adm Policy Ment Health. 2019;46(2):255–70.

45. Vasileiou K, Barnett J, Thorpe S, Young T. Characterising and justifying sample size sufficiency in interview-based studies: systematic analysis of qualitative health research over a 15-year period. BMC Med Res Methodol. 2018 Dec 21;18(1):148.

46. Guetterman TC, Fetters MD, Creswell JW. Integrating Quantitative and Qualitative Results in Health Science Mixed Methods Research Through Joint Displays. Ann Fam Med. 2015 Nov 1;13(6):554–61.

47. Roberts NP, Kitchiner NJ, Kenardy J, et al. Early psychological intervention following recent trauma: A systematic review and meta-analysis. Eur J Psychotraumatol. 2019 Dec 31;10(1):1695486.

48. National Institute for Health and Care Excellence. Clinical guideline (CG83) Rehabilitation after critical illness in adults. March 2009. Accessed 14/10/2023:https://www.nice.org.uk/Guidance/CG83

For peer review only

For peer review only - http://bmjopen.bmj.com/site/about/guidelines.xhtml

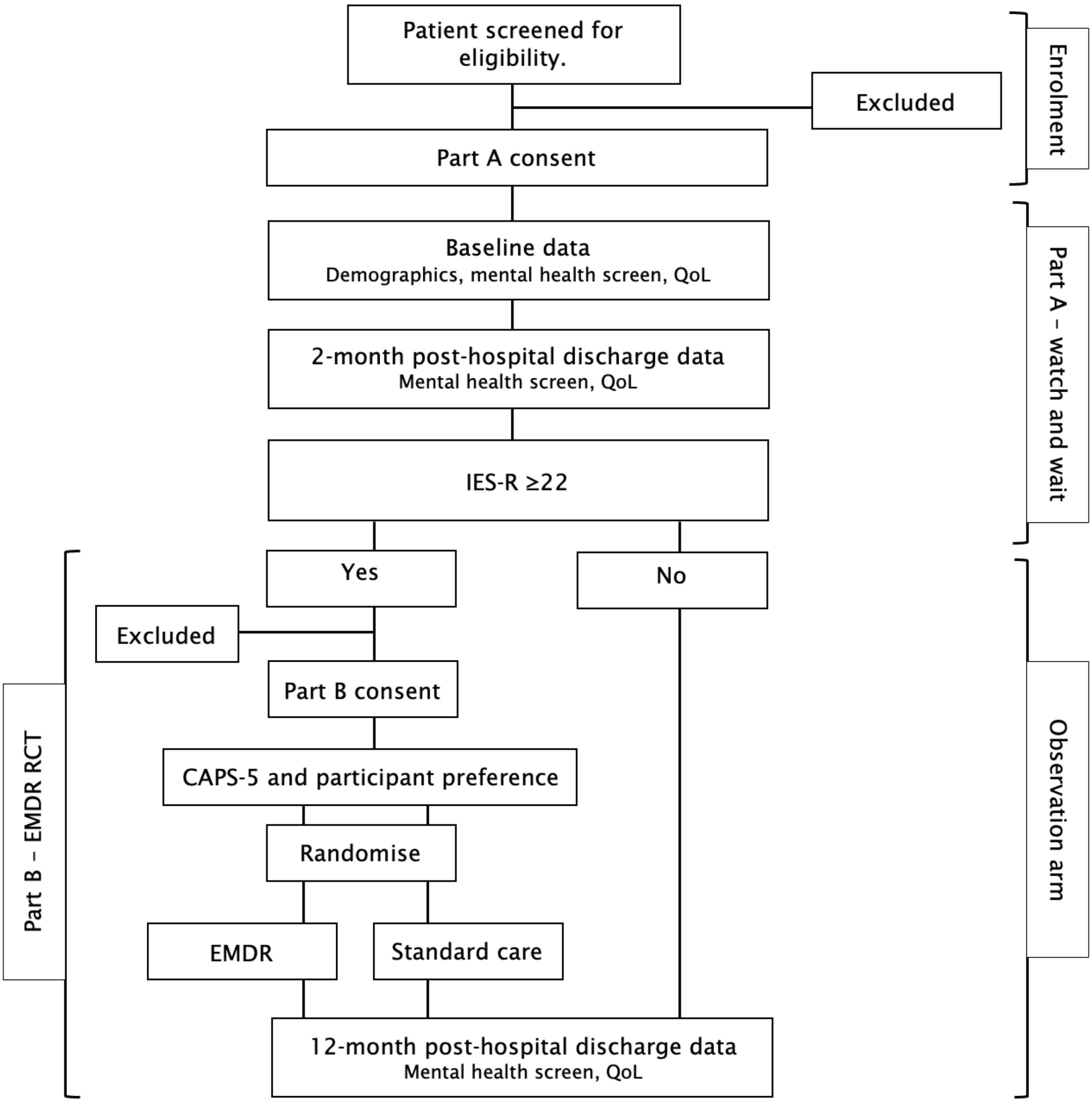
Patient screened for eligibility.
Excluded
Part A consent
Enrolment
Baseline data
Demographics, mental health screen, QoL
2-month post-hospital discharge data
Mental health screen, QoL
IES-R ≥22
Part A – watch and wait
Yes
No
Excluded
Part B consent
CAPS-5 and participant preference
Randomise
EMDR
Standard care
12-month post-hospital discharge data
Mental health screen, QoL
Part B – EMDR RCT
Observation arm

Supplemental file 1: Reporting checklist for protocol of a clinical trial.

Based on the SPIRIT guidelines.

Instructions to authors

Complete this checklist by entering the page numbers from your manuscript where readers will find each of the items listed below.

Your article may not currently address all the items on the checklist. Please modify your text to include the missing information. If you are certain that an item does not apply, please write "n/a" and provide a short explanation.

Upload your completed checklist as an extra file when you submit to a journal.

In your methods section, say that you used the SPIRITreporting guidelines, and cite them as:

Chan A-W, Tetzlaff JM, Gøtzsche PC, Altman DG, Mann H, Berlin J, Dickersin K, Hróbjartsson A, Schulz KF, Parulekar WR, Krleža-Jerić K, Laupacis A, Moher D. SPIRIT 2013 Explanation and Elaboration: Guidance for protocols of clinical trials. BMJ. 2013;346:e7586

		Reporting Item	Page Number
**Administrative information**			
Title	#1	Descriptive title identifying the study design, population, interventions, and, if applicable, trial acronym	1
Trial registration	#2a	Trial identifier and registry name. If not yet registered, name of intended registry	2
Trial registration: data set	#2b	All items from the World Health Organization Trial Registration Data Set	2 (link in text)
Protocol version	#3	Date and version identifier	n/a
Funding	#4	Sources and types of financial, material, and other support	1

For peer review only

Roles and responsibilities: contributorship	#5a	Names, affiliations, and roles of protocol contributors	1 and 13
Roles and responsibilities: sponsor contact information	#5b	Name and contact information for the trial sponsor	n/a
Roles and responsibilities: sponsor and funder	#5c	Role of study sponsor and funders, if any, in study design; collection, management, analysis, and interpretation of data; writing of the report; and the decision to submit the report for publication, including whether they will have ultimate authority over any of these activities	n/a
Roles and responsibilities: committees	#5d	Composition, roles, and responsibilities of the coordinating centre, steering committee, endpoint adjudication committee, data management team, and other individuals or groups overseeing the trial, if applicable (see Item 21a for data monitoring committee)	n/a
**Introduction**			
Background and rationale	#6a	Description of research question and justification for undertaking the trial, including summary of relevant studies (published and unpublished) examining benefits and harms for each intervention	2
Background and rationale: choice of comparators	#6b	Explanation for choice of comparators	2
Objectives	#7	Specific objectives or hypotheses	3
Trial design	#8	Description of trial design including type of trial (eg, parallel group, crossover, factorial, single group), allocation ratio, and framework (eg,	3

For peer review only

		superiority, equivalence, non-inferiority, exploratory)	
**Methods: Participants, interventions, and outcomes**			
Study setting	#9	Description of study settings (eg, community clinic, academic hospital) and list of countries where data will be collected. Reference to where list of study sites can be obtained	4
Eligibility criteria	#10	Inclusion and exclusion criteria for participants. If applicable, eligibility criteria for study centres and individuals who will perform the interventions (eg, surgeons, psychotherapists)	4
Interventions: description	#11a	Interventions for each group with sufficient detail to allow replication, including how and when they will be administered	8 and supplementary material
Interventions: modifications	#11b	Criteria for discontinuing or modifying allocated interventions for a given trial participant (eg, drug dose change in response to harms, participant request, or improving / worsening disease)	8
Interventions: adherance	#11c	Strategies to improve adherence to intervention protocols, and any procedures for monitoring adherence (eg, drug tablet return; laboratory tests)	8 and supplementary material
Interventions: concomitant care	#11d	Relevant concomitant care and interventions that are permitted or prohibited during the trial	8
Outcomes	#12	Primary, secondary, and other outcomes, including the specific measurement variable (eg, systolic blood pressure), analysis metric (eg, change from baseline, final value, time to event), method of aggregation (eg, median, proportion), and time point for each outcome. Explanation of the clinical relevance of chosen efficacy and harm outcomes is strongly recommended	9

For peer review only

Participant timeline	#13	Time schedule of enrolment, interventions (including any run-ins and washouts), assessments, and visits for participants. A schematic diagram is highly recommended (see Figure)	see figure 1
Sample size	#14	Estimated number of participants needed to achieve study objectives and how it was determined, including clinical and statistical assumptions supporting any sample size calculations	10
Recruitment	#15	Strategies for achieving adequate participant enrolment to reach target sample size	5 and 8
**Methods: Assignment of interventions (for controlled trials)**			
Allocation: sequence generation	#16a	Method of generating the allocation sequence (eg, computer-generated random numbers), and list of any factors for stratification. To reduce predictability of a random sequence, details of any planned restriction (eg, blocking) should be provided in a separate document that is unavailable to those who enrol participants or assign interventions	8
Allocation concealment mechanism	#16b	Mechanism of implementing the allocation sequence (eg, central telephone; sequentially numbered, opaque, sealed envelopes), describing any steps to conceal the sequence until interventions are assigned	8
Allocation: implementation	#16c	Who will generate the allocation sequence, who will enrol participants, and who will assign participants to interventions	8

For peer review only

Blinding (masking)	#17a	Who will be blinded after assignment to interventions (eg, trial participants, care providers, outcome assessors, data analysts), and how	8
Blinding (masking): emergency unblinding	#17b	If blinded, circumstances under which unblinding is permissible, and procedure for revealing a participant's allocated intervention during the trial	11
**Methods: Data collection, management, and analysis**			
Data collection plan	#18a	Plans for assessment and collection of outcome, baseline, and other trial data, including any related processes to promote data quality (eg, duplicate measurements, training of assessors) and a description of study instruments (eg, questionnaires, laboratory tests) along with their reliability and validity, if known. Reference to where data collection forms can be found, if not in the protocol	9, 10, 11
Data collection plan: retention	#18b	Plans to promote participant retention and complete follow-up, including list of any outcome data to be collected for participants who discontinue or deviate from intervention protocols	10,11
Data management	#19	Plans for data entry, coding, security, and storage, including any related processes to promote data quality (eg, double data entry; range checks for data values). Reference to where details of data management procedures can be found, if not in the protocol	9
Statistics: outcomes	#20a	Statistical methods for analysing primary and secondary outcomes. Reference to where other details of the statistical analysis plan can be found, if not in the protocol	10

For peer review only

Statistics: additional analyses	#20b	Methods for any additional analyses (eg, subgroup and adjusted analyses)	na
Statistics: analysis population and missing data	#20c	Definition of analysis population relating to protocol non-adherence (eg, as randomised analysis), and any statistical methods to handle missing data (eg, multiple imputation)	na
**Methods: Monitoring**			
Data monitoring: formal committee	#21a	Composition of data monitoring committee (DMC); summary of its role and reporting structure; statement of whether it is independent from the sponsor and competing interests; and reference to where further details about its charter can be found, if not in the protocol. Alternatively, an explanation of why a DMC is not needed	12
Data monitoring: interim analysis	#21b	Description of any interim analyses and stopping guidelines, including who will have access to these interim results and make the final decision to terminate the trial	12
Harms	#22	Plans for collecting, assessing, reporting, and managing solicited and spontaneously reported adverse events and other unintended effects of trial interventions or trial conduct	11
Auditing	#23	Frequency and procedures for auditing trial conduct, if any, and whether the process will be independent from investigators and the sponsor	12
**Ethics and dissemination**			
Research ethics approval	#24	Plans for seeking research ethics committee / institutional review board (REC / IRB) approval	12
Protocol amendments	#25	Plans for communicating important protocol modifications (eg, changes to eligibility criteria, outcomes, analyses) to relevant parties (eg,	12

For peer review only

		investigators, REC / IRBs, trial participants, trial registries, journals, regulators)	
Consent or assent	#26a	Who will obtain informed consent or assent from potential trial participants or authorised surrogates, and how (see Item 32)	5
Consent or assent: ancillary studies	#26b	Additional consent provisions for collection and use of participant data and biological specimens in ancillary studies, if applicable	na
Confidentiality	#27	How personal information about potential and enrolled participants will be collected, shared, and maintained in order to protect confidentiality before, during, and after the trial	9
Declaration of interests	#28	Financial and other competing interests for principal investigators for the overall trial and each study site	15
Data access	#29	Statement of who will have access to the final trial dataset, and disclosure of contractual agreements that limit such access for investigators	10
Ancillary and post trial care	#30	Provisions, if any, for ancillary and post-trial care, and for compensation to those who suffer harm from trial participation	11
Dissemination policy: trial results	#31a	Plans for investigators and sponsor to communicate trial results to participants, healthcare professionals, the public, and other relevant groups (eg, via publication, reporting in results databases, or other data sharing arrangements), including any publication restrictions	12
Dissemination policy: authorship	#31b	Authorship eligibility guidelines and any intended use of professional writers	12
Dissemination policy: reproducible research	#31c	Plans, if any, for granting public access to the full protocol, participant-level dataset, and statistical code	12

For peer review only

Appendices

Informed consent materials	#32	Model consent form and other related documentation given to participants and authorised surrogates	3
Biological specimens	#33	Plans for collection, laboratory evaluation, and storage of biological specimens for genetic or molecular analysis in the current trial and for future use in ancillary studies, if applicable	na

Notes:

- 2b: 2 (link in text)
- 11a: 8 and supplementary material
- 11c: 8 and supplementary material
- 13: see figure 1 The SPIRIT Explanation and Elaboration paper is distributed under the terms of the Creative Commons Attribution License CC-BY-NC. This checklist was completed on 09. November 2023 using https://www.goodreports.org/, a tool made by the EQUATOR Network in collaboration with Penelope.ai

For peer review only

Supplementary file 2. EMDR treatment protocol reported according to TIDieR (Template for intervention description and replication)

Why: EMDR (Eye Movement Desensitization and Reprocessing) is hypothesised to alleviate post-traumatic symptoms among patients discharged from intensive care units (ICUs), by facilitating the adaptive processing of traumatic memories. The bilateral stimulation involved in EMDR is thought to assist in integrating memories of distressing experiences, potentially reducing the impact of trauma on mental health recovery. This study will investigate the feasibility and acceptability of delivering a randomised controlled trial (RCT) of EMDR following discharge from ICU.

What (material): No physical or informational materials were used during the intervention.

What (procedures): EMDR is a protocolised talking therapy which consists of 8 phases:

Phase 1: History taking and treatment planning: discuss participant history, with identification of traumatic events, develop a treatment plan, and assess participant's internal and external resources.

Phase 2: **Preparation:** establish a therapeutic alliance through explanation of EMDR process, discuss expectations, concerns, and questions, and equip participant with techniques to address disturbance that may arise.

Phase 3: **Assessment:** identify a target event, including associated memories, feelings, and images. Ask the participant to rate the associated disturbance, from zero to ten, using the Subjective Units of Distress scale, (SUD) and the Validity of Cognition (VOC) scale.

Phase 4: **Desensitisation:** focussing on the target event, the participant will be asked to perform side-to-side eye movements, tapping or sounds. This phase will be repeated until SUD reduces to zero or one.

Phase 5: **Installation:** Once SUD has reduced to zero-one, the participant will be guided to associate a positive belief, with the target event, until it feels consistently true.

Phase 6: **Body scan:** the participant is guided to hold both the target event and positive belief in mind, while scanning their bodily sensations from head to toe. If they identify lingering disturbance, they will repeat phase 4, until reprocessing is complete.

Phases 7 and 8 are delivered at the end of each session and are designed to ensure safety.

Phase 7: Closure: The psychological therapist assists the participant to return to a state of calm.

Phase 8: Re-evaluation: The psychological therapist and EMDR (Eye-movement desensitisation and reprocessing)

participants discuss recently processed memories and identify future target memories and directions for treatment.

Who provided: EMDR was delivered by trained, experienced psychological therapists employed by the United Kingdom (UK) National Health Service (NHS). The therapists are undergoing monthly peer to peer support and are being supervised by an EMDR Europe accredited Consultant Clinical Psychologist.

How (mode of delivery; individual or group): EMDR is delivered face-to-face or via Internet teleconference, according to participant preference.

Where: Face-to-face sessions will take place within the NHS psychological therapies clinic. Online teleconference will take place via Microsoft Teams™. Where participants are unable to attend either face-to-face or Internet sessions then a tablet with Internet dongle will be provided by the study team.

When and how much: Sessions will be delivered weekly, last for up to 60 minutes, and are provided individually. Participants will receive up to 16 sessions of EMDR.

Tailoring: The nature of trauma focused psychological therapies necessitates a personalised approach to the intervention. However representative sample of sessions will be recorded and reviewed by an expert practitioner for fidelity using the EMDR Fidelity rating scale.

How well (planned): Adherence to EMDR intervention will be expressed as a percentage of sessions offered against sessions completed. Psychological therapists will complete a diary card which will be made available to the study team at the end of the intervention.

For peer review only

Supplemental file 3. Secondary clinical outcome measures – description and timing

		Timepoint		
**Measure**	**Description**	**Baseline**	**Two-months post-hospital discharge**	**12-months post-hospital discharge**
**Impact of Events Scale-Revised (IES-R)** (1)	IES-R is a 22-question patient reported outcome measure (PROM) widely used to assess symptoms of PTSD in critical care research(2,3), recommended by critical care core outcome dataset developers(4–6) and the International Conference of Harmonisation of Outcome Measures(7). The 22 questions cover symptoms of intrusion, avoidance, and hyperarousal. Participants indicate how distressing the symptoms have been over the last 7 days. Symptom severity can be 0 (not at all), 1 (a little bit), 2 (moderately), 3 (quite a bit), 4 (extremely), giving a total scoring range of 0 to 88.<br>A range of cut-offs for diagnosing PTSD have been identified in different populations. To maximise sensitivity, and minimise risk of leaving PTSD untreated, we will apply the lower cut-off of 22 and retrospectively conduct a sensitivity analysis against the CAPS-5.	X	X	X
**Clinician Administered PTSD Scale for DSM-5 (CAPS-5)**(8)	CAPS-5 is a structured diagnostic interview, considered the gold-standard assessment of PTSD symptoms. In addition to evaluating the 20 symptoms listed in the DSM-5, the questions focus on the onset and duration of these symptoms, the subjective distress experienced, how these symptoms impact an individual's social and occupational functioning, any improvement in symptoms since a prior CAPS assessment, the overall validity of the responses, the severity of PTSD as a whole, and the criteria for the dissociative subtype, which encompasses depersonalization and derealization.<br>CAPS-5 assessment will be conducted face-to-face or over the phone, (according to participant preference) methods which deliver comparable results.		X (participants who have consented to part B only)	X (participants who have consented to part B only)
**Clinical Global Impression-Severity scale (CGI-S)**(9)	The CGI-S can be used to assess symptom severity and response to treatment. It requires a clinician to rate the severity of a patient's mental illness, on a seven-point scale ranging from; 1 – normal, not at all ill, 2 – borderline mentally ill, 3 – mildly ill, 4 – moderately ill, 5 – markedly ill, 6 – severely ill, 7 – among the most extremely ill patients.		X (participants who have consented to part B only)	X (participants who have consented to part B only)

For peer review only

**Clinical Global Impression-Improvement scale (CGI-I)**(9)	The CGI-I requires a clinician to assess degree of improvement since baseline, in a participant's symptoms, on a seven-point ranging from; 1-very much improved; 2-much improved; 3-minimally improved; 4-no change; 5-minimally worse; 6-much worse; 7-very much worse.		X (participants who have consented to part B only)	X (participants who have consented to part B only)
**Patient Health Questionnaire (PHQ-9)**(10)	Self-administred, vaildated tool assesses depressive symptom severity(10–12). Scores are calculated by assigning 0 for 'not at all, 1-'several days', 2 - 'more than half the days' or 3 – 'nearly every day' for responses to nine questions, giving a score in the range 0-27. PHQ-9 score of 0-4 demonstrates no - minimal depression severity. 5-9 = mild severity, 10-14 = moderate severity, 15-19 = moderately severe, 20-27 = severe.	X	X	X
**Generalised Anxiety Disorder-7 (GAD-7)**(13)	Seven-question, self-administered tool is validated to assess for anxiety symptom severity. Scores are calculated by assigning 0 for 'not at all, 1-'several days', 2 - 'more than half the days' or 3 – 'nearly every day' for responses to nine questions, giving a score in the range 0-21. GAD-7 scores of 5, 10 and 15 represent cut-offs for mild, moderate and severe anxiety respectively.	X	X	X
**Health Related Quality of Life: Euroqual 5-level 5-Dimension (EQ-5D-5L)**(14)	Comprises five quality-of-life dimensions; mobility, self-care, usual activities, pain/discomfort and anxiety/depression. Participants report levels ranging from 'no problems' to 'extreme problems'.	X	X	X

For peer review only

EMERALD – Participant Interview Guide – Intervention group

Introduction and orientation:

- Thank you for agreeing to take part in this research and for being interviewed today.
- Cover logistics of video conference interview and outline the plan if technology issues are experienced.
- Discuss recording and how we will store and use the information in this interview.
- The interview will cover a range of questions about your experiences. There are no right or wrong answers. We are very interested in your experience of the study as it will help us to design better studies in the future.
- If at any time you do not wish to answer a question or are unsure how to answer, that's okay.
- If you have any opinions that may seem challenging or critical, then that is okay too.

- Do you have any questions about this?
- Can I start the recording now?

Semi-structured interview questions:

**Introduction**	Can you talk me through how you became aware of the EMERALD study and run through your involvement?
**1. Affective Attitude:**<br><br>*How an individual feels about the intervention.*	I'd like you to think about how it felt taking part in the study.<br><br>• How did you feel towards EMDR? What informed that feeling?<br>• What did you like (or dislike) about EMDR? What were the best/worst parts?<br>• Enquire about feelings of calmness, positivity, discomfort, anxiety, feelings of panic etc; may need to probe for more information with appropriate reflective listening.
**2. Burden:**<br><br>*The amount of effort that was required to participate in the intervention*	I would like to discuss how much effort it took for you to undertake the EMERALD study – including any perceived difficulties or challenges?<br><br>• Did you experience any practical problems – online or face-to-face?<br>• Were there any consequences of receiving EMDR for you?<br>• What was the impact on your daily life?<br>• **Prompts:** may include cost, money, time commitment, or emotional burden<br>• If not yet addressed: What about other members of your household or family? (How did they support you if needed?)<br>• **Prompts:** help with internet access, use of technology, time, transport, financial
**3. Ethicality:**<br><br>*The extent to which the intervention has good fit*	I would like to explore the ethics of EMDR, such as respect, competence, responsibility, and integrity.<br><br>• Do you think there are any ethical issues with any aspect of taking part in the study?

For peer review only

with an individual's value system	• Can you describe any ethical implications to using EMDR in wider practice?<br>• Was there anything we could have done to make the study fairer?<br>• **Prompt:** In what ways do you think having EMDR fair or not fair?
**4. Intervention Coherence:**<br><br>*The extent to which the participant understands the intervention and how it works*	I notice that you attended XX sessions out of YY sessions arranged. I'd like to talk about your understanding of the EMDR.<br><br>• Having had EMDR, how do you think it helped or (doesn't/didn't help) regarding your symptoms of post-traumatic stress?<br>• How do you think it might work or not work?<br>• How much did you feel that EMDR was the right approach?<br>• What are your thoughts on the number of sessions? Do you think attending more (or fewer) sessions would change how effective it was?
**5. Opportunity Cost:**<br><br>*Experienced opportunity cost: The benefits, profits or values that were given up to engage in the intervention*	I'd like you to describe your feelings of the value and the potential costs of undertaking EMDR.<br><br>• What were the pros and cons of EMDR? Was there anything that you particularly liked or disliked?<br>• Was there anything that you had to give up so that you could have your EMDR?<br>• Do you have any reservations that you would like to discuss?
**6. Perceived Effectiveness:**<br><br>*The extent to which the intervention is perceived to have achieved its intended purpose.*	• How effective do/did you think (engaging with) EMDR was?<br>• How has EMDR affected the things that are important to you?<br>• **Prompts:** What weren't you able to do prior to EMDR that was important to you?<br>• Are you able to do this now? (work, home, social relationships)<br>• In what ways do you feel better/worse, emotionally, or physically?
**7. Self-efficacy:**<br><br>*The participant's confidence that they can perform the behaviour(s) required to participate in the intervention*	• How confident were you that you could (safely) take part in the study +/- the EMDR?<br>• How easy or difficult was it to stay engaged/concentrate for the whole session?<br>• **Prompt:** did it stir up any unpleasant or pleasant emotions?<br>• Do you think **you** had an ability to benefit?<br>• How did you address any challenges that we have previously discussed?

Question:
When considering all the things you've spoken about, what would be your overall summary of taking part in EMERALD?

Is there anything that you think could be done better?
Is there anything else you'd like to tell us?

Thank you for giving me your time again today and thank you for taking part in our study.

For peer review only

EMERALD – Psychological therapist Interview Guide

Introduction and orientation

Semi-structured interview questions:

**Introduction**	Can you talk me through how you became aware of the EMERALD study and run through your involvement?<br>I'd like you to consider the study group meetings, referrals, and delivery of the EMDR.
**1. Affective Attitude:**<br><br>*How an individual feels about the intervention.*	How did it feel to be taking part in the study.<br><br>**Prompts:**<br>Emotionally, did you enjoy it?<br><br>Enquire about anything that you found surprising, uncomfortable, or anxiety provoking; may need to probe for more information with appropriate reflective listening.
**2. Burden:**<br><br>*The amount of effort that was required to participate in the intervention*	I would like to discuss how much effort you feel it took to undertake the EMERALD study – you perception – any difficulties or challenges?<br><br>Did you experience any practical problems –online or face-to-face, burden of the additional workload?<br><br>What was the impact on your working life – time commitment and emotional strain.<br><br>**Prompt:** could include cost, money, time/workload, or emotional burden<br><br>Did you experience any (other) burden(s) because of your involvement?<br><br>**Prompt:** help with internet access, financial
**3. Ethicality:**<br><br>*The extent to which the intervention has good fit with an individual's value system*	Do you think there are any ethical issues with any aspect of the study?<br><br>What about the randomisation, do you think there are ethical issues some people getting or not getting EMDR when traumatised?<br><br>**Prompt:** In what ways do you think having EMDR or not having EMDR is fair or not fair?<br><br>Was there anything we could have done to make the study fairer?

For peer review only

**4. Intervention Coherence:**<br><br>*The extent to which the participant understands the intervention and how it works*	What is your understanding of EMDR and how it may be applicable with these participants?<br><br>What do think was the aim of the EMDR?<br><br>Did it seem sensible to use for post-ICU traumatic stress?<br><br>How might it work for these patients?<br><br>Do you think attending more (or fewer) sessions would change how effective it was?
**5. Opportunity Cost:**<br><br>*Experienced opportunity cost: The benefits, profits or values that were given up to engage in the intervention*	Could you describe your feelings of the value of undertaking EMDR?<br><br>**Prompt:** do you think this was better or worse than alternatives, including the option of doing nothing?<br><br>Do you have any reservations that you would like to discuss?
**6. Perceived Effectiveness:**<br><br>*The extent to which the intervention is perceived to have achieved its intended purpose.*	Do you think that EMDR has been effective for your participants?<br><br>**Prompt:** How do you feel it may have affected various aspects of their life? (work, home, social relationships)<br><br>Do you think they feel better, emotionally, or physically?
**7. Self-efficacy:**<br><br>*The participant's confidence that they can perform the behaviour(s) required to participate in the intervention*	How confident were you that you could deliver the study/EMDR as per the protocol?<br><br>**Prompt:** did it stir up any unpleasant emotions?<br><br>Do you think **you** were able to benefit?<br><br>How did you address any challenges that we have previously discussed?

We are very near to the end of our interview today and I would like to hear about how you felt overall.

Question:
When considering all the things you've spoken about, what would be your overall summary of taking part in EMERALD?

Is there anything that you think could be done better?

Is there anything else you'd like to tell us?

Thank you for giving me your time again today and thank you for taking part in our study.

For peer review only
