# Supplementary material for: Mixed-methods randomised study exploring the feasibility and acceptability of eye-movement desensitisation and reprocessing for improving the mental health of traumatised survivors of intensive care following hospital discharge: protocol
Source: BMJ Open. 2024 Jan 29;14(1):e081969. doi: 10.1136/bmjopen-2023-081969 (PMC10826543; doi:10.1136/bmjopen-2023-081969)
Supplement: Reviewer comments [file bmjopen-2023-081969.reviewer_comments.pdf]

PEER REVIEW HISTORY

BMJ Open publishes all reviews undertaken for accepted manuscripts. Reviewers are asked to complete a checklist review form (**http://bmjopen.bmj.com/site/about/resources/checklist.pdf**) and are provided with free text boxes to elaborate on their assessment. These free text comments are reproduced below.

ARTICLE DETAILS

**TITLE (PROVISIONAL)**	Protocol for a mixed methods randomised study exploring the feasibility and acceptability of Eye-Movement Desensitisation and Reprocessing for improving the mental health of traumatised survivors of intensive care following hospital discharge.
**AUTHORS**	Bates, Andrew; Golding, Hannah; Rushbrook, Sophie; Highfield, Julie; Pattison, Natalie; Baldwin, David; Grocott, Michael P. W.; Cusack, Rebecca

This article was not externally reviewed at BMJ Open. Protocols that have been independently assessed prior to submission to BMJ Open are usually fast-tracked to publication on the grounds that further substantial changes will not be possible. This independent assessment will usually be external, independent review for both a substantial grant award from a non-commercial or government funder and ethics approval.
